# Biocrusts intensify water redistribution and improve water availability to dryland vegetation: insights from a spatially-explicit ecohydrological model

**DOI:** 10.3389/fmicb.2023.1179291

**Published:** 2023-06-27

**Authors:** Selina Baldauf, Yolanda Cantón, Britta Tietjen

**Affiliations:** ^1^Institute of Biology, Theoretical Ecology, Freie Universität Berlin, Berlin, Germany; ^2^Department of Agronomy, University of Almería, Almería, Spain; ^3^Research Centre for Scientific Collections from the University of Almería (CECOUAL), Almería, Spain; ^4^Berlin-Brandenburg Institute of Advanced Biodiversity Research (BBIB), Berlin, Germany

**Keywords:** biocrust, biological soil crust, dryland, ecohydrology, process-based model, soil moisture, runoff, landscape model

## Abstract

Biocrusts are ecosystem engineers in drylands and structure the landscape through their ecohydrological effects. They regulate soil infiltration and evaporation but also surface water redistribution, providing important resources for vascular vegetation. Spatially-explicit ecohydrological models are useful tools to explore such ecohydrological mechanisms, but biocrusts have rarely been included in them. We contribute to closing this gap and assess how biocrusts shape spatio-temporal water fluxes and availability in a dryland landscape and how landscape hydrology is affected by climate-change induced shifts in the biocrust community. We extended the spatially-explicit, process-based ecohydrological dryland model EcoHyD by a biocrust layer which modifies water in- and outputs from the soil and affects surface runoff. The model was parameterized for a dryland hillslope in South-East Spain using field and literature data. We assessed the effect of biocrusts on landscape-scale soil moisture distribution, plant-available water and the hydrological processes behind it. To quantify the biocrust effects, we ran the model with and without biocrusts for a wet and dry year. Finally, we compared the effect of incipient and well-developed cyanobacteria- and lichen biocrusts on surface hydrology to evaluate possible paths forward if biocrust communities change due to climate change. Our model reproduced the runoff source-sink patterns typical of the landscape. The spatial differentiation of soil moisture in deeper layers matched the observed distribution of vascular vegetation. Biocrusts in the model led to higher water availability overall and in vegetated areas of the landscape and that this positive effect in part also held for a dry year. Compared to bare soil and incipient biocrusts, well-developed biocrusts protected the soil from evaporation thus preserving soil moisture despite lower infiltration while at the same time redistributing water toward downhill vegetation. Biocrust cover is vital for water redistribution and plant-available water but potential changes of biocrust composition and cover can reduce their ability of being a water source and sustaining dryland vegetation. The process-based model used in this study is a promising tool to further quantify and assess long-term scenarios of climate change and how it affects ecohydrological feedbacks that shape and stabilize dryland landscapes.

## 1. Introduction

Semiarid landscapes are often characterized by a patchy mosaic of vascular vegetation and bare interplant spaces which are covered by a continuous cover of biocrusts, communities of poikilohydric organisms such as lichens, cyanobacteria and mosses (Thiéry et al., 1995; Ludwig et al., 2005; Weber et al., 2016). Biocrusts grow on and within the first few centimeters of the soil and act as a boundary layer between the soil and the atmosphere mediating most water inputs and outputs from the soil (Belnap et al., 2001; Belnap and Büdel, 2016). A general emerging pattern in drylands worldwide is that they decrease soil water infiltration, while at the same time conserving the moisture in the upper soil layers (Eldridge et al., 2020) for example by reducing evaporation (Chamizo et al., 2013a) or by increasing water holding capacity (Sun et al., 2022; Shi et al., 2023). Local interactions between biocrusts and hydrological processes have a cascading effect on the landscape-scale redistribution of rainfall water via surface runoff (Cantón et al., 2011; Chamizo et al., 2012a; Guan and Cao, 2019; Kidron, 2021). Thus, biocrusts have been described as an “organizing principle” (Weber et al., 2016) in drylands, because they support the formation of islands of fertility (Weber et al., 2016), where nutrient-rich runon water can infiltrate and benefit vascular plants (Thiéry et al., 1995; Ludwig et al., 2005; Belnap and Büdel, 2016; Rodríguez-Caballero et al., 2018a).

The exact mechanisms behind biocrust effects on hydrological processes are complex and context dependent (Chamizo et al., 2016a; Eldridge et al., 2020) and depend on the cover and the species composition of the crust. High moss cover and an advanced developmental stage of the biocrust usually increase soil infiltration and reduce runoff from the biocrust due to high surface roughness and macroporosity (Miralles-Mellado et al., 2011; Belnap et al., 2013). In contrast, some biocrust lichens have hydrophobic surfaces (Kidron et al., 1999; Souza-Egipsy et al., 2002; Pintado et al., 2005), which inhibit water uptake and therefore reduce soil infiltration and increase surface runoff (Cantón et al., 2002; Souza-Egipsy et al., 2002; Rodríguez-Caballero et al., 2013). The direct effect of species composition on hydrological processes interacts with other factors such as precipitation magnitude and intensity. For example, biocrusts can decrease runoff during low intensity rainfall events, but this effect can disappear for more intense and high magnitude events that saturate the soil and the biocrust layer (Chamizo et al., 2012a,b; Rodríguez-Caballero et al., 2014a). Moreover, environmental conditions such as the soil type play an important role in governing the effect of crusts on hydrology (Warren, 2003; Chamizo et al., 2013b). Biocrusts on sandy soils tend to decrease infiltration compared to uncrusted soils, whereas the effects on fine-textured soils are less clear or opposite (Warren, 2003; Eldridge et al., 2020). Indirectly, biocrusts can also regulate the movement of water in the soil by altering soil properties such as porosity and aggregation or surface water storage (reviewed in Chamizo et al., 2016a). In addition, it is important to consider the spatial scale when evaluating the effect of biocrusts on hydrology, because the connectivity between retentive (e.g., vascular vegetation) and conductive (e.g., biocrusts or bare soil) landscape elements ultimately determines landscape-scale runoff (Ludwig et al., 2005).

In light of these effects, biocrusts are vital for ecosystem functioning, especially considering that climate change reduces the overall water availability (Huang et al., 2017; Cramer et al., 2018) and changes individual rainfall characteristics toward less frequent but more extreme events in many drylands (Toreti et al., 2013; Toreti and Naveau, 2015; IPCC, 2021). Under such conditions, an intact network of biocrust areas can help to buffer drought years and provide vital water and nutrient input for vascular vegetation growth (Rodríguez-Caballero et al., 2018a; Antoninka et al., 2020). At the same time, such a network can reduce or even prevent land degradation by erosion following high intensity and magnitude rainfall events (Belnap and Büdel, 2016; Chamizo et al., 2016b). Biocrusts are thus vitally important to mitigate climate change impacts in dryland ecosystems. However, climate change and other disturbances also affect the biocrusts themselves, reducing their species richness (Ladrón de Guevara et al., 2018), functional diversity (Mallen-Cooper et al., 2018), and ultimately their overall cover (Baldauf et al., 2021; Finger-Higgens et al., 2022). Increased temperatures are linked to shifts from late-successional lichen- and moss-dominated biocrusts to early-successional cyanobacteria-dominated biocrusts and biocrust lichens are particularly affected, with significant reductions in coverage reported from both long-term climate manipulation experiments and field observations (Escolar et al., 2012; Ladrón de Guevara et al., 2014; Ferrenberg et al., 2015; Ladrón de Guevara et al., 2018; Finger-Higgens et al., 2022). Such changes in biocrusts threaten the complex interactions and feedbacks between biocrust and vegetated areas in drylands, which help to sustain the landscape’s productivity. It is therefore vital to gain a better understanding of the feedbacks between biocrusts and landscape hydrology.

To date, most studies on the interaction between biocrusts and hydrology have been field studies conducted on spatial scales below 10 m^2^ with many of these studies focusing on scales below 0.05 m^2^ (Eldridge et al., 2020). But small-scale effects cannot necessarily be extrapolated and quantified on the landscape scale (Chamizo et al., 2016a), as important processes such as the runoff-runon network on the hillslope are not captured (Rodríguez-Caballero et al., 2014a). To address this gap and to improve predictions, previous studies called for including biocrusts into ecohydrological and hydrological models (e.g., Rodríguez-Caballero et al., 2015a; Hui et al., 2021). Such models have long been used to study the interactions between local and landscape-scale hydrological processes and their effect on vegetation in drylands (e.g., Franz et al., 2010; Tietjen et al., 2010; Tietjen, 2015). However, only few models explicitly account for biocrusts (Cantón et al., 2002; Rodríguez-Caballero et al., 2015a; Whitney et al., 2017; Chen et al., 2018, 2019; Jia et al., 2019). These models either focus on the time scale of single rainfall events rather than on longer time scales (Cantón et al., 2002; Rodríguez-Caballero et al., 2015a) or they are not spatially explicit and can therefore only capture point scale processes (Whitney et al., 2017; Chen et al., 2018, 2019; Jia et al., 2019). Also, only few of the existing approaches distinguish between different biocrust types (Whitney et al., 2017; Chen et al., 2019). This limits their potential to examine the impacts of biocrusts on the landscape water balance and the ecohydrological feedbacks under climate change conditions. Spatially-explicit ecohydrological simulation models have a large potential to address these limitations and to complement experimental and observational studies.

In this study, we aim to understand the effect of different biocrusts on landscape-scale soil moisture as a result of their role in water redistribution via runoff, soil evaporation and infiltration. We extended the spatially-explicit, process-based ecohydrological simulation model EcoHyD (Tietjen et al., 2009, 2010) by including a biocrust layer that affects several hydrological processes in the model. We calibrated and validated the model with field measurements from the El Cautivo site in South-East Spain and evaluated the impact of different biocrust types on water redistribution under current and dry climatic conditions. In particular, we addressed the following questions for the El Cautivo study site:

- What are the main processes shaping spatio-temporal soil moisture patterns and plant available water in the El Cautivo landscape under current conditions?
- What is the quantitative contribution of biocrusts to landscape-scale soil moisture in wet and dry years and which are the major processes involved?
- How will landscape-scale soil moisture and hydrological processes be affected by a climate change-induced shift from well-developed lichen to incipient cyanobacteria biocrusts?

## 2. Materials and methods

### 2.1. Site description

**Figure 1:**
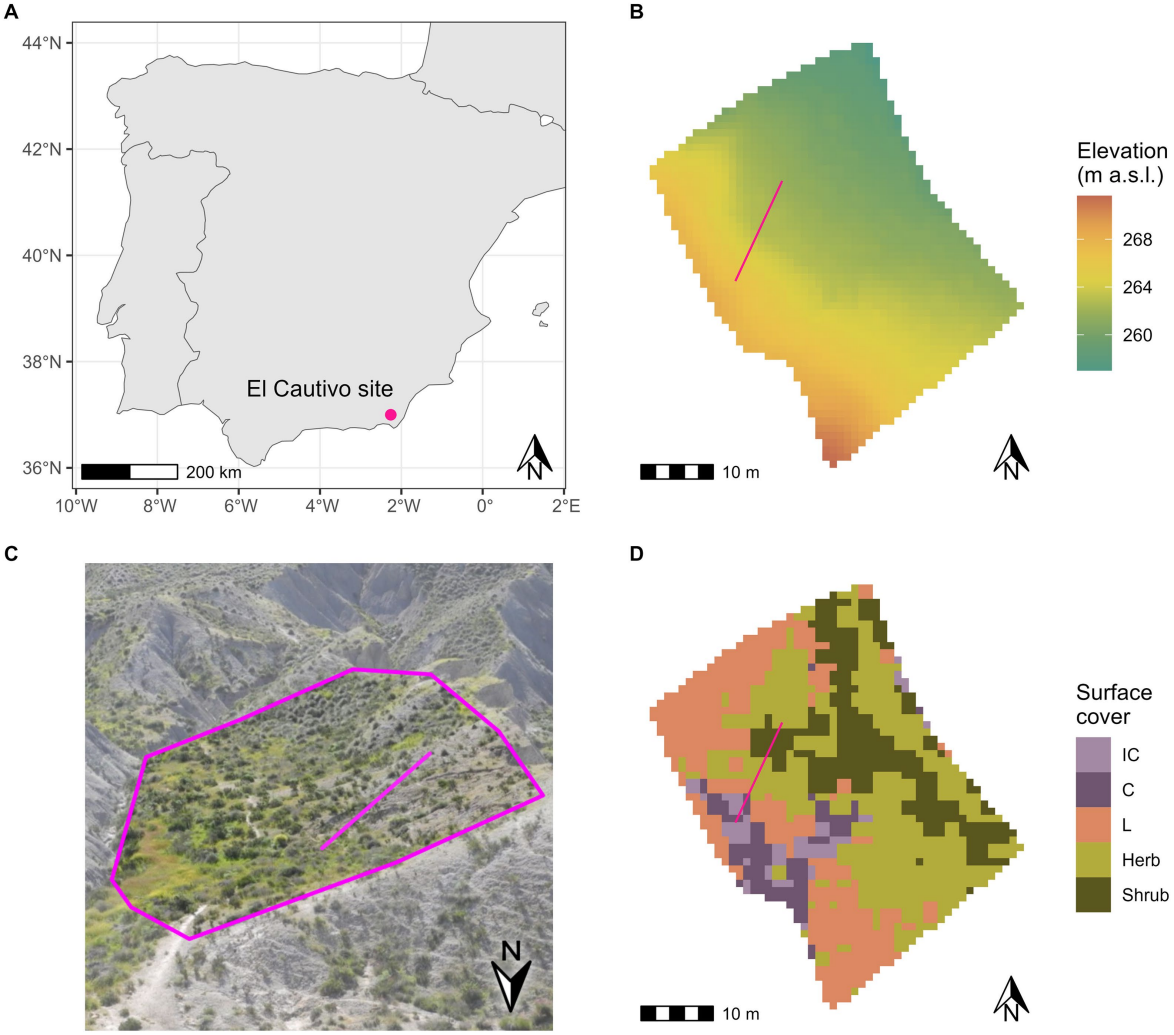
Location and characteristics of the El-Cautivo study site in the Tabernas badlands. **(A)** Location of the study site in South-East Spain. **(B)** Elevation map of the region (1 m resolution). **(C)** Photo of the study site. The pink polygon roughly encloses the hillslope investigated in this study (Photo: Selina Baldauf, 03/2017). Please note that the orientation in this photo is different from the maps (see north arrow). **(D)** Surface cover map of the El Cautivo hillslope. Vascular vegetation (Herb, herbaceous annual vegetation, shrubs) and biocrust (IC, incipient cyanobacteria; C, cyanobacteria; L, lichen biocrust) cover map from hyperspectral imaging conducted in 2010. The pink line in b-d shows the transect for which modeling results are analyzed in detail.

The El Cautivo site is located in the badlands of the Tabernas desert in South-East Spain (37°0′N, 2°26′W, 200 m a.s.l., Figure 1A). The climate is semi-arid Mediterranean with a mean annual temperature of 17.8°C at the Tabernas weather station, ranging from 10.3°C in January to 27.0°C in August. The mean annual precipitation is 235 mm, with a highly seasonal rainfall with monthly values between 2 mm in July to 29 mm in November (Agencia Estatal de Meteorología (AEMET), 2020). In this study, we concentrated on one north-east-facing hillslope in the area (Figures 1B,C). On this hillslope, the vegetation distribution is mainly controlled by topography: biocrusts cover the steep upper parts of the hillslope and vascular vegetation, consisting of annual herbaceous plants (e.g., *Stipa capensis, Plantago ovata*, and *Bromus rubens*) and shrubs (mainly dwarf shrub such as *Hammada articulata, Artemisia barrilieri*, and *Salsola genistoides*), covers the lower and less steep parts of the hillslope (Figure 1D; Cantón et al., 2004). A detailed site description of El Cautivo is provided in Rodríguez-Caballero et al. (2013).

There are three different types of biocrust on the hillslope: incipient cyanobacteria, cyanobacteria and lichen biocrust. The incipient cyanobacteria biocrust represents the first successional stage of biocrust formation and is characterized by a low to very low biomass density of cyanobacteria and is very thin and light-colored. They are dominated by filamentous non-heterocystous cyanobacteria (60.5%) like the bundle-forming *Microcoleus vaginatus* and *Microcoleus steenstrupii* but also contain heterocystous forms such as *Nostoc commune* and unicellular and colonial groups like *Chroococcidiopsis* ssp. The well-developed cyanobacteria biocrust forms as cyanobacteria biomass increases and it is associated with some pioneer lichens and characterized by a darker color. In this biocrust, the filamentous non-heterocystous cyanobacteria get replaced by unicellular and colonial cyanobacteria (50.6%) with heterocystous cyanobacteria like *Scytonema hyalinum* and *Nostoc commune* still being found in this later stage of biocrust succession (Roncero-Ramos et al., 2020). The lichen biocrust on the hillslope is dominated by light-colored lichens, mainly *Squamarina lentigera* and *Diploschistes diacapsis.*

### 2.2. Model description

We used the spatially-explicit process-based ecohydrological dryland model EcoHyD (Tietjen et al., 2009, 2010). The model consists of a hydrological (Tietjen et al., 2009) and a vegetation sub-model (Tietjen et al., 2010; Lohmann et al., 2012) that calculate processes for every grid cell of the landscape in two soil layers. We extended the model and added a biocrust layer that affects all hydrological processes except transpiration in the model. In the extended model, grid cells can be covered by vascular vegetation (annual herbaceous or shrubs) and different biocrust types. For simplicity, each grid cell can only be covered by one biocrust type and one type of vascular vegetation. Vascular vegetation and biocrusts can co-occur in the same grid cell. The simulated grid consists of a square of 74 × 74 grid cells of 1 m resolution (total extent *ca.* 5,500 m^2^).

**Figure 2:**
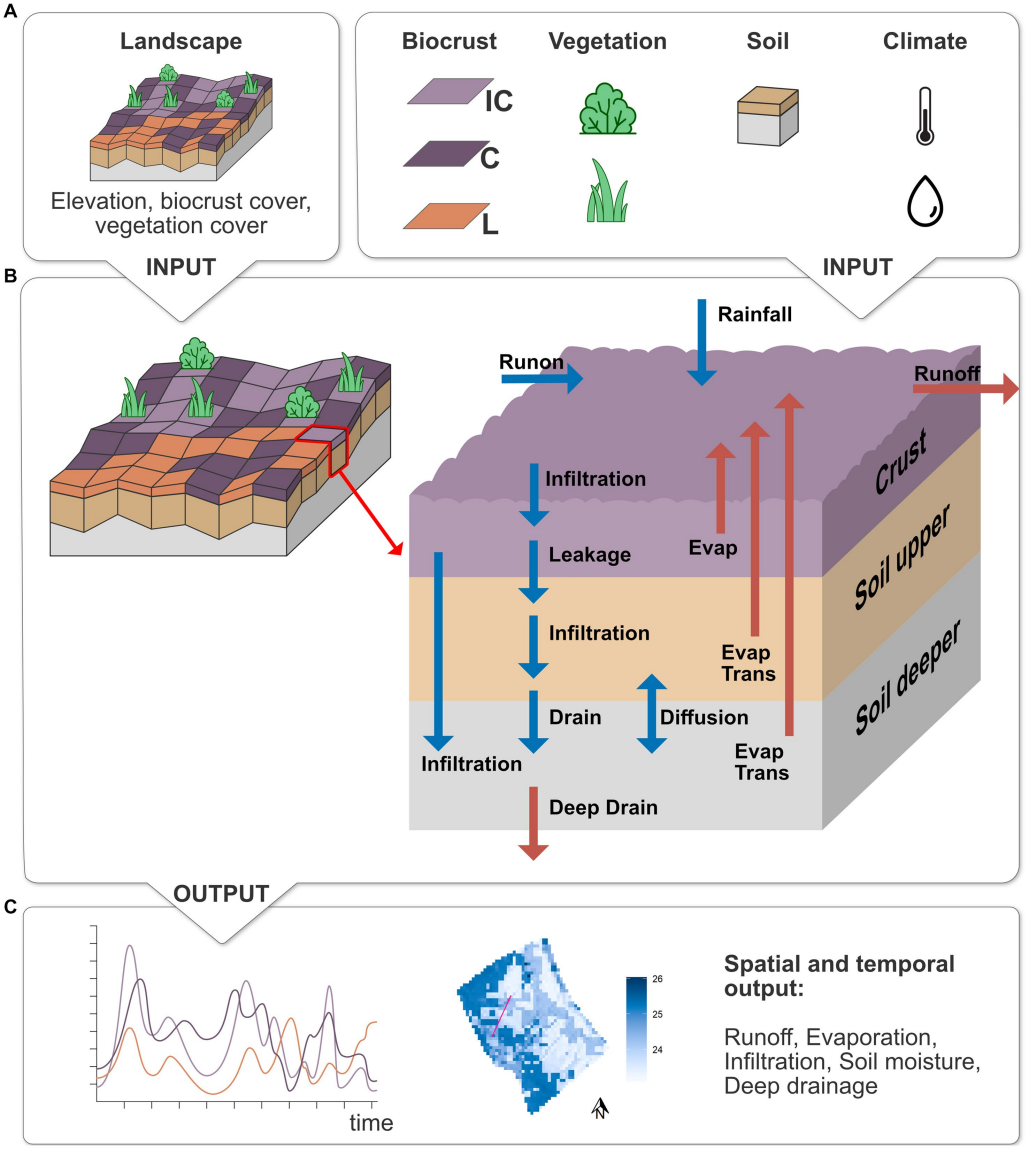
Overview of the extended EcoHyD model with biocrust layer. **(A)** Required model input: spatial maps of elevation, biocrust and vegetation cover, as well as parameters for biocrusts (IC, incipient cyanobacteria; C, cyanobacteria; L, lichen), vegetation (shrubs and annual herbaceous vegetation), soil and climate (temperature and rainfall). **(B)** Model processes in one grid cell for the 3 layers. Blue arrows show the processes that provide water input into a cell, red arrows show water output. **(C)** Model output that is analyzed in this study: temporal output of different processes for each of the biocrust cover types and spatial output for each grid cell.

The processes of the biocrust layer were implemented based on the one-dimensional ecohydrological biocrust model by Whitney et al. (2017). In the following, we will give a brief summary of the model and Figure 2 shows a conceptual overview of the model with all model input, processes and outputs that were analyzed in this study. A more detailed process description of the model with all the changes that were made to the original model can be found in the Supplementary material.

The hydrological sub-model of EcoHyD calculates the water dynamics of two soil layers in each grid cell on an hourly time step and was extended to include biocrusts as a layer on top of the first soil layer (see Figure 2). The biocrust layer modulates all water input and output from the soil. First, rainfall is transformed into surface water from where it can infiltrate into the biocrust layer, depending on its porosity and current moisture. If the biocrust type that covers the grid cell is hydrophobic, biocrust infiltration is reduced by a hydrophobicity factor which becomes higher as the biocrust becomes dryer. After the biocrust is saturated, the remaining surface water leaks through the biocrust into the upper soil layer where it infiltrates following a Green and Ampt (1911) approach. As soon as the upper soil layer is saturated (i.e., its water content reaches field capacity), water drains from the upper into the deeper soil layer. When the deeper soil layer is saturated, water drains into deeper layers that are not explicitly simulated in our model and can be interpreted as deep soil infiltration or groundwater recharge, depending on the thickness of the simulated soil layers. The remaining surface water that has not infiltrated into the soil is redistributed in the landscape via surface runoff. Each grid cell passes runoff to its lowest neighboring cell. The amount of runoff depends on the amount of surface water, the slope and the surface roughness of the respective cell. Vascular vegetation reduces runoff maximally by factor 0.5 for 100% vegetation cover (Tietjen et al., 2009). Biocrusts, on the other hand, increase runoff by a factor of 3.6 (constrained by the availability of surface water). This factor is based on measurements from the study site that showed that runoff from biocrusted soils is on average 3.6 times higher than from non-crusted soils (Cantón et al., 2001, 2002). Biocrust evaporation and soil evapotranspiration are evaluated once at the end of each day based on the mean, minimum and maximum temperature of that day. Water from the biocrust layer can evaporate if the biocrust moisture is above the biocrust-specific hygroscopic point. Soil evapotranspiration depends on the vegetation cover of the cell as well as the biocrust specific evaporation reduction factor. In this study, we simulated a one-year time span and assumed the vegetation cover to remain constant throughout the simulation. Therefore, all processes in the vegetation sub-model (e.g., vegetation growth and dispersal) were switched off. Vascular vegetation cover was set at the beginning of the simulation according to observed cover from the field.

### 2.3. Model parameterization

The model requires maps with the elevation and the surface cover of each grid cell as well as vascular vegetation, biocrust and soil parameters and hourly time series of temperature and rainfall as input (Figure 2). We used field data from the study site for model parameterization whenever possible. If field data was unavailable, we used values from literature and calibrated remaining parameters using soil moisture measurements from the field. Below we describe the model parameterization in detail.

#### 2.3.1. Elevation and surface cover maps

We used a digital elevation map of the region with a 1 × 1 m^2^ resolution that was built from elevation points acquired from an airborne LiDAR survey (resolution: 4 points per m2) [see Rodríguez-Caballero et al. (2015a) for more details] to parameterize the elevation of the grid cells (Figure 1B). To parameterize surface cover, we used a surface cover map obtained by classification of a hyperspectral image taken of the hillslope in 2010 (Rodríguez-Caballero et al., 2014b). The resolution of this surface cover map was increased from 1.5 × 1.5 m^2^ to 1 × 1 m^2^ using the nearest neighbor interpolation method to match the resolution of the digital elevation map. The surface cover of the hillslope consists of five classes (Figure 1D): Shrubs (20% of hillslope cells), annual herbaceous vegetation (40% of hillslope cells), lichen (39.5% of hillslope cells), cyanobacteria (6.5% of hillslope cells) and incipient cyanobacteria biocrust (4% of hillslope cells).

Every cell covered by vascular vegetation was assigned a constant cover value of 80% of the respective vegetation type, cells covered by biocrusts were assigned a 100% biocrust cover. For simplicity, we assumed that herbaceous plants and shrubs do not occur in the same cell. Representing the conditions in the field, all vegetated cells were additionally covered by 100% cyanobacteria biocrusts leaving no bare ground on the hillslope. The resulting landscape data consists of a square of 74 × 74 grid cells (*ca.* 5,500 m^2^). Of these, we only selected those cells that were part of the hillslope shown in Figures 1B–D for the analysis of model results.

#### 2.3.2. Climate data

We used climate data from an on-site weather station for the hydrological year 2009–2010 (October 2009–September 2010). Temperature and rainfall data were aggregated from 30 min (rain) and 10 min (temperature) to 1 h intervals by calculating the mean temperature and the sum of rainfall. We filled one missing temperature value by linear approximation between the two nearest measurements. In the rainfall time series, 504 values were missing. We replaced the missing values with 0 because a linear approximation would have increased annual rainfall by 76 mm. The hydrological year 2009–2010 is already wet in comparison to the long-term average (MAP of Tabernas 235 mm) and we wanted to avoid artificially inflating the total rainfall of this year. We compared the daily rainfall time series with values from the Tabernas weather station, which is around 10 km away [Red de Información Agroclimática de Andalucía (RIA), 2023]. This comparison showed that our method of filling missing values did not change the rainfall pattern and amounts (Supplementary Figure S1A). We reordered the climate time series to start in June, because the EcoHyD model starts simulations in the dry season. The climate time series used as model input had a mean annual temperature of 19.5°C and an annual rainfall sum of 375 mm (Supplementary Figure S1B).

#### 2.3.3. Soil and biocrust parameters

A summary of all soil and biocrust parameters can be found in Supplementary Tables S1–S3. When possible, we used field and literature data on biocrust and soil characteristics to parameterize the model. However, many soil and biocrust parameters were unknown or values were variable between studies, so we first conducted a sensitivity analysis to determine those parameters that had the highest influence on modeled soil moisture. Afterwards, we calibrated these parameters to best match observed patterns of soil moisture. For this, we obtained hourly soil moisture data measured in two depths (3 cm and 10 cm) under incipient cyanobacteria, cyanobacteria and lichen biocrusts for the same period as the climate data (Chamizo et al., 2016c).

##### 2.3.3.1. Sensitivity analysis

We tested the sensitivity of the fit of modeled to measured soil moisture data (root mean square error (RMSE)) for ten biocrust and four soil parameters in the months of December 2009 to January 2010 (see Supplementary Table S1 for details on tested parameters). This period covered the start of the wet season with a large increase in soil moisture, as well as some weeks of the wet season with generally high soil moisture. We tested parameter value ranges that appeared realistic from the literature research (see Supplementary Table S1 for references). As the soil moisture measurements were conducted in a flat part of the hill, we simulated a flat area for the sensitivity analysis. Sensitivity analysis was conducted using a modified version of the Morris method of elementary effects (Campolongo et al., 2007) as implemented in the “morris” function of the R package “sensitivity” (Iooss et al., 2021). The most sensitive parameters (both in terms of direct and interactive effect) were the four biocrust parameters thickness, porosity, saturated hydraulic conductivity and evaporation reduction factor as well as the four soil parameters saturated hydraulic conductivity, field capacity, suction at the wetting front and wilting point (See Supplementary Figure S2).

##### 2.3.3.2. Calibration

These most sensitive parameters were then calibrated within the same value range and for the same time period used for the sensitivity analysis (Supplementary Table S1). Calibration was performed with a genetic differential evolution algorithm procedure using the “DEoptim” R package (Ardia et al., 2011; Mullen et al., 2011). The target of the calibration function was the RMSE of modeled and measured soil moisture in the two soil layers. Biocrust parameters were calibrated separately for the three biocrust types and soil parameters were calibrated to be the same underneath all biocrust types. The calibration results are shown together with the parameterization in Supplementary Tables S2, S3. The calibrated model could reproduce the soil moisture dynamics in the two soil layers reasonably well for the entire climate time series (overall RMSE upper layer: 3.2–3.4%, deeper layer 3.1–3.6%, Supplementary Figure S3).

##### 2.3.3.3. Other parameters

Biocrust parameters that were not calibrated, were taken from parameter values for different biocrust roughness classes reported in Whitney et al. (2017). We used the parameter values of the first (lowest) roughness class for the incipient cyanobacteria biocrust, the second for the cyanobacteria and third for the lichen biocrust because generally, biocrust roughness increases with its developmental stage (Caster et al., 2021). The biocrust hydrophobicity function in the model was parameterized using results from water drop penetration tests conducted on different biocrust samples from El Cautivo. We used quadratic fits of water drop penetration time depending on biocrust moisture. The results of the water drop penetration tests showed a large difference between biocrusts (Supplementary Figure S4). In a dry lichen biocrust, the water took up to 300 min to be absorbed whereas it took below 2 min in the other biocrust types. Considering the 1 h model time step, we included hydrophobicity only for the lichen biocrust (hydrophobicity parameters see Supplementary Table S3).

#### 2.3.4. Vegetation parameters

Not all vegetation parameters usually included in EcoHyD were relevant for this study, as vegetation cover was assumed to be constant (see above). The only relevant vegetation parameters were parameters on the shading effect of plants on evaporation and the relationship between aboveground cover and belowground root fractions, impacting water losses by transpiration. Since we did not have measured values from El Cautivo for these parameters, we used the standard values for shrubs and grasses as described in Lohmann et al. (2012). To represent the herbaceous annual vegetation, we used the parameter values for perennial grasses. The reason for this is that in this study we simulate a very thin upper layer of 6 cm that is exceeded by the roots of annual vegetation, which has not been implemented in previous model versions. Representing our herbaceous vegetation by perennial grasses, allows for a deeper rooting system and does not affect other results because we only simulate the period of one year.

### 2.4. Simulation experiments

We ran several simulation experiments to answer our research questions.

First, we established a baseline scenario in which we simulated the El Cautivo site under current conditions with biocrust cover (scenario “baseline-biocrust”). For this baseline-biocrust scenario, we used the parameterization described above. We then looked at water redistribution, evaporation, deep drainage and runoff in each grid cell to evaluate how water is distributed in the landscape and how uphill areas covered with biocrusts affect downhill soil moisture. We evaluated deep drainage as an estimate for plant available water in the soil layers below the thin upper soil layers that we simulated explicitly. We analyzed the spatial distribution patterns of these processes both in the hillslope landscape and in a selected transect that contained both uphill biocrusts and downhill vegetation (see Figures 1B,D).

Second, we wanted to compare the effects of biocrusts in a wet and a dry year. For this, we removed all biocrust cover from the landscape while leaving the vascular vegetation and ran the model with bare soil, as it is originally represented in EcoHyD (scenario “no-biocrust”). We ran these two scenarios (baseline-biocrust and no-biocrust) for a wet year (rainfall from 2010) and a 50% reduced rainfall time series (every rainfall event of 2010–50%). We evaluated if biocrusts could sustain a higher soil moisture in dry years compared to soils not covered by biocrusts. To look at the processes in detail, we also compared water availability and fluxes in the selected hillslope transect between the two scenarios for a wet and a dry year.

Third, we compared the separate effects of the three different biocrust types (incipient cyanobacteria, cyanobacteria and lichen) to assess how a likely change from well-developed to incipient biocrusts under climate change affects soil water availability and processes. For this, we conducted three simulations, in which the landscape was covered by only one of the three biocrust types, respectively. Vascular vegetation was the same as in the baseline-biocrust scenario. We then compared landscape mean values of infiltration, evapotranspiration, runoff, deep drainage and soil moisture between the landscapes covered by the different biocrust types. With this scenario we wanted to get an estimate of how landscape hydrology and plant available water could change if the biocrust cover shifted from well-developed lichen and cyanobacteria toward incipient cyanobacteria biocrusts due to climate or land-use change.

## 3. Results

### 3.1. Soil moisture patterns and plant-available water under current conditions

**Figure 3:**
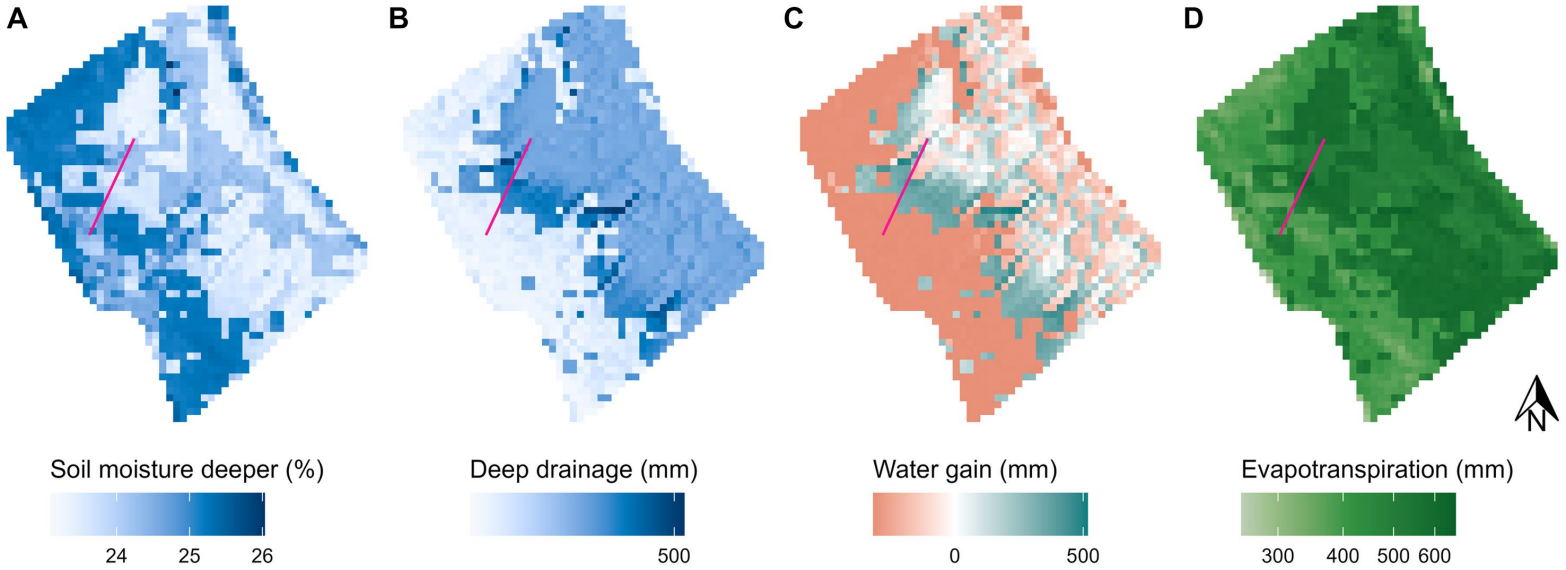
Spatial distribution of hydrological variables and processes on the El Cautivo hillslope in January. **(A)** Mean monthly soil moisture in the lower soil layer (6–20 cm). **(B)** Monthly sum of deep drainage to deep soil layers (below 20 cm). **(C)** Monthly sum of water gain (i.e., runon – runoff). **(D)** Monthly sum of evapotranspiration from both soil layers. The pink line shows the hillslope transect that was selected for further detailed spatio-temporal analysis. It crosses a biocrusted section at the top and a vegetated section further down the hillslope (see also Figure 1).

The baseline-biocrust scenario showed that soil moisture and hydrological processes in the wet winter months are governed by the interaction between hillslope steepness and surface cover (Figure 3; additional processes and other months are given in the Supplementary Figures S5–S8). Soil moisture in the upper layer (0–6 cm) was similar across the hillslope but slightly lower under incipient cyanobacteria biocrusts and highest under lichen biocrusts (Supplementary Figure S6A). In the deeper soil layer (6–20 cm), biocrusted and vegetated areas showed clear differences (Figure 3A): soil moisture was highest under lichen biocrusts and lowest under annual vascular vegetation, while areas covered by incipient cyanobacteria biocrusts were in between. Deep drainage (i.e., plant-available water below 20 cm of soil depth) showed the opposite differentiation (Figure 3B): it was higher under vascular vegetation and lower under biocrusts. The highest deep drainage values were observed in the middle part of the hillslope in the transition zones between uphill biocrusts and downhill vascular vegetation. Redistributing water via surface runoff can result in either positive (i.e., runon higher than runoff) or negative (i.e., runoff higher than runon) water gain (Figure 3C). The middle part of the hillslope gained water, while the steep uphill areas covered with biocrusts lost water. This additional runoff provided more water to the middle part of the hillslope where it drained to layers below the deeper soil layer. Evapotranspiration from both soil layers was higher under vegetation, especially annual herbaceous vegetation, and lower under biocrusts (Figure 1D).

**Figure 4:**
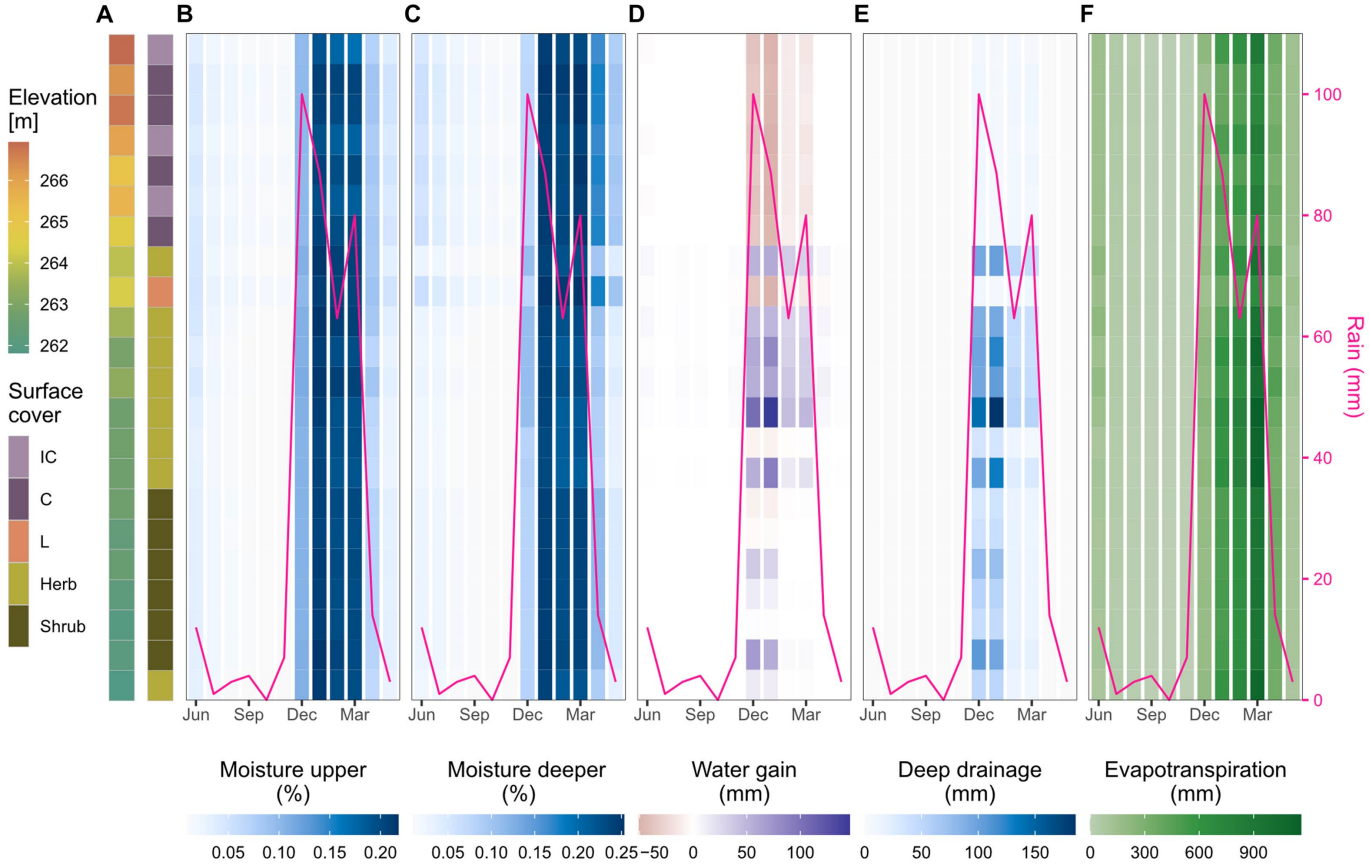
Water availability and fluxes along the selected hillslope transect. **(A)** Elevation and surface cover of the transect cells selected for the spatio-temporal analysis. See also Figures 1B–D for location of the transect in the hillslope. **(B–F)** Spatio-temporal development of soil moisture, water gain, deep drainage and evapotranspiration from both soil layers in the cells of the selected hillslope transect. The figures show monthly mean moisture and monthly sum of the hydrological processes for each transect cell (*y*-axis) over the course of one year (*x*-axis).

The hillslope transect analysis (Figure 4A) showed a similar spatial differentiation of hydrological processes and soil moisture throughout the year. At the beginning of the wet season with the first rainfall in December, soil moisture in both layers increased in all parts of the transect (Figures 4B,C). Water gains were particularly high in the first vegetated cells (Figure 4D) where runoff from the upper biocrusted meters infiltrated and drained to deeper layers (Figure 4E). Water redistribution was only observed in the wet season (December to March) and was most pronounced in December and January when rainfall was high. In these 2 months, water was redistributed from the upper, biocrusted cells to the vegetation cells of the whole transect. In February and March, water redistribution was limited to the upper vegetated meters of the transect and the lower part did not receive additional water anymore. While water redistribution and deep drainage were only observed until March and mainly in the middle part of the transect, soil moisture remained elevated until April and May, especially in the biocrusted upper part. Evapotranspiration was highest in the wet season and it increased with increasing temperatures until March (Figure 4F). In April, soil moisture and evapotranspiration sharply declined and the dry season began. In the summer months, there was almost no soil water available for hydrological processes. Soil drying was faster in the upper soil layer and in the vegetated lower part of the transect.

### 3.2. Quantification of biocrust effects in a wet and dry year

**Figure 5:**
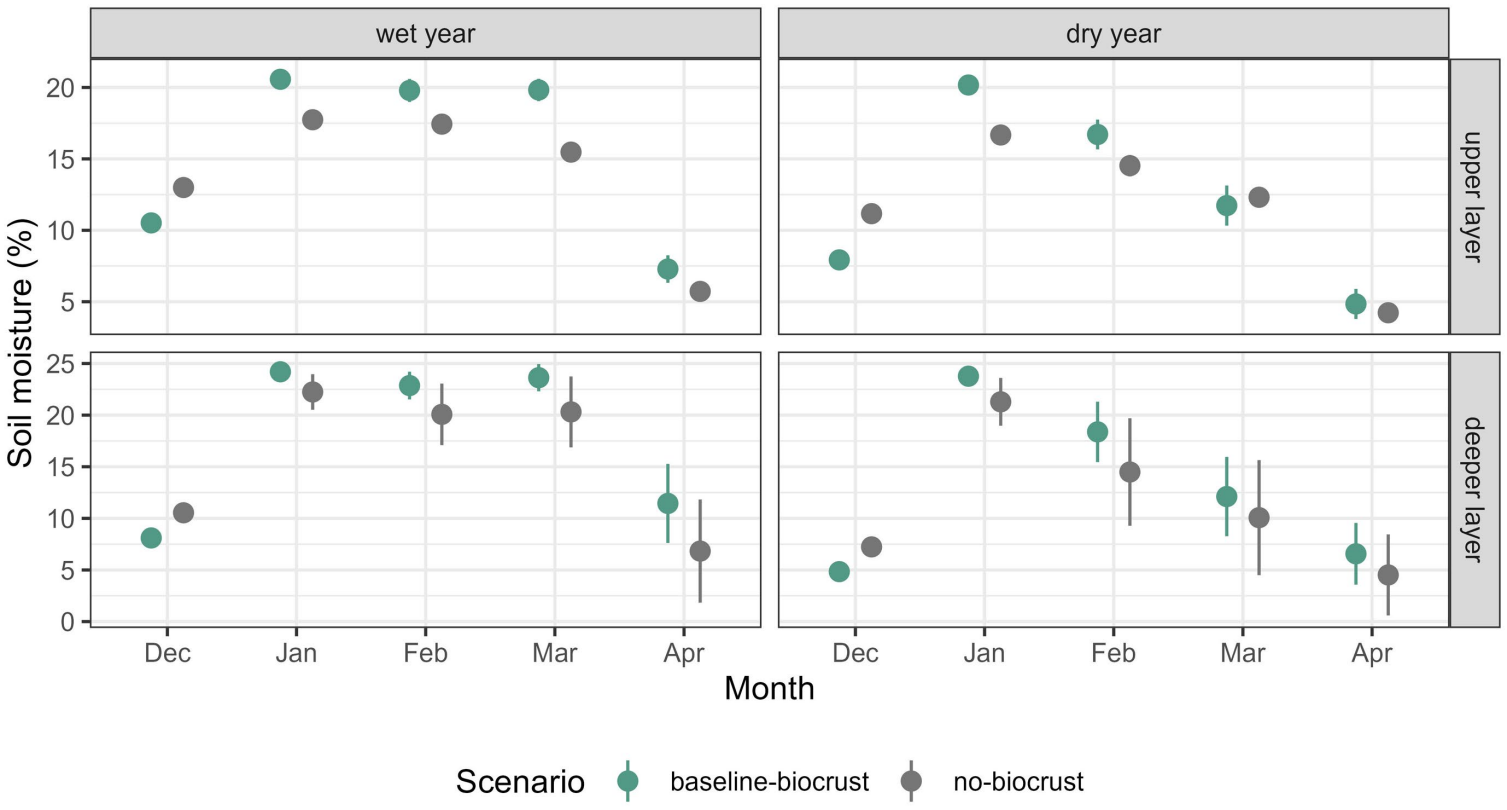
Effect of biocrusts on soil moisture in a wet and a dry year. Comparison of median ± standard deviation of mean monthly soil moisture in the upper and lower soil layer of all grid cells of the hillslope between the baseline-biocrust scenario with current biocrust cover and the no-biocrust scenario without biocrust cover. The first column shows the distribution under current climate conditions and the second column shows the soil moisture distribution for a dry climate with 50% reduced rainfall.

To evaluate the effects of biocrusts, we compared soil moisture between the baseline-biocrust and the no-biocrust scenario for a wet and a dry year. In the wet year, soil moisture in both layers was higher in the baseline-biocrust scenario, except for the month of December (Figure 5, see Supplementary Figure S9 for all months). In the deeper layer, soil moisture was more similar between the baseline-biocrust and no-biocrust scenarios with a tendency for higher soil moisture in the presence of biocrusts. Moreover, soil moisture variability in the deeper layer was higher without biocrusts. In the dry year, soil moisture was generally lower compared to the wet year, except for January when it was similar. The differences between the scenarios with and without biocrusts accentuated. While soil moisture was higher without biocrusts in both soil layers in December, afterwards it was mostly higher with biocrusts. Soil moisture in the deeper layer was highly variable from February on, and soil moisture was more similar in the scenarios with and without biocrusts.

To assess the effects of biocrusts on water availability and fluxes in a wet and a dry year, we analyzed the differences between the baseline-biocrust and the no-biocrust scenario in the selected hillslope transect throughout one year (Supplementary Figure S10). We saw that biocrusts generally led to more runoff from the non-vegetated part of the hillslope with several implications: Although soil moisture in the upper layer was higher during the wet season due to lower losses by evaporation, soil moisture in the deeper layer and deep drainage were reduced under biocrusts compared to bare soil. At the same time, the higher runoff from biocrusts provided more water for the vascular vegetation in the lower part of the hillslope. These biocrust effects on water availability and fluxes were similar for the wet and the dry year.

### 3.3. Difference in soil moisture and hydrological processes between well-developed and incipient biocrust

**Figure 6:**
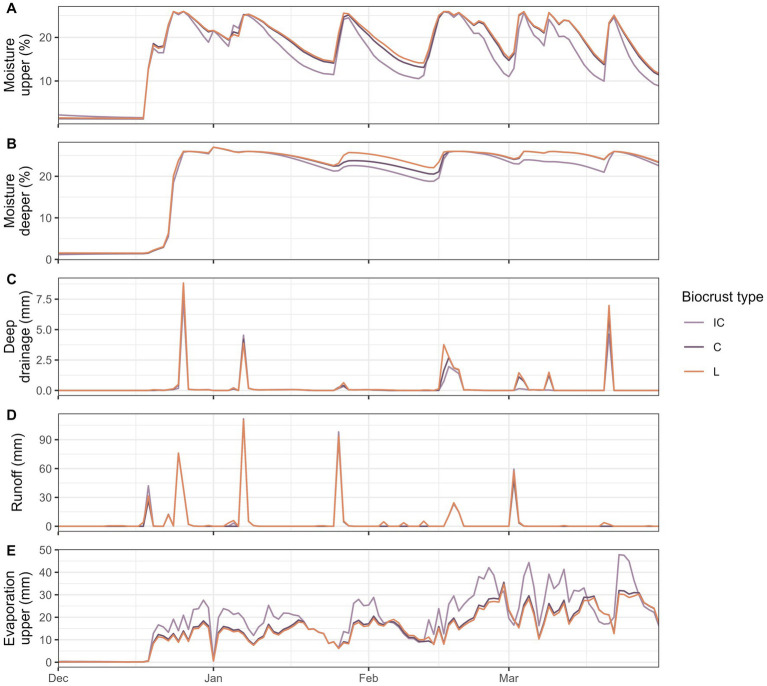
Comparison of the effect of different biocrust types (IC, incipient cyanobacteria; C, cyanobacteria; L, lichen) on soil moisture and water fluxes. Simulated daily mean soil moisture **(A,B)** and sum of hydrological processes **(C–E)** in the wet season from December until April. Please note that the mean values presented here were only calculated for grid cells that were not covered by vascular vegetation in order to get a comparison between the three biocrust types.

A substantial difference in soil moisture between the scenarios with the three different biocrust types was only observed in the winter season. At the start of the wet season in January, soil moisture was similar below all biocrust types (Figures 6A,B). In the following months, soil moisture was higher below well-developed cyanobacteria and lichen biocrusts in both layers mainly due to the different soil drying curves: Soil moisture was similar below all biocrusts following a rainfall event, but then the soil dried faster under incipient cyanobacteria biocrusts. Soil moisture was also more variable in the upper and more constant in the deeper layer. Deep drainage was higher under well-developed biocrusts, especially lichens (Figure 6C). On some days in March, deep drainage was only observed under well-developed biocrusts but not under incipient cyanobacteria. The highest overall runoff was observed in December and January when soil moisture in the upper layer was near saturation and rainfall was high (Figure 6D). The three biocrust types had similar runoff patterns, but lichens had slightly higher runoff compared to the other types. For some small rainfall events in February and March only lichen biocrusts triggered runoff. Grid cells covered with incipient cyanobacteria biocrusts lost more water through evaporation from the upper layer compared to cells covered with lichen or well-developed cyanobacteria biocrust (Figure 6E).

## 4. Discussion

### 4.1. Soil moisture patterns and plant-available water under current conditions

The spatio-temporal patterns of modeled soil moisture and hydrological processes reveal the interactive effects of topography, surface cover and rainfall. Soil moisture until 20 cm depth was mainly driven by seasonal patterns of rainfall and evapotranspiration, while the distribution of water in deeper soil layers was determined by water redistribution in the landscape.

The soil moisture distribution in the upper layer did not show a clear difference between areas with and without vascular vegetation, probably because the thin layer (6 cm) saturated quickly during large precipitation events irrespective of surface cover (Supplementary Figure S6A). In the deeper layer, soil moisture showed a clear differentiation by surface cover, which was mainly driven by differences in evapotranspiration that was higher under vegetated areas and incipient cyanobacteria. The daily timestep for evaporation in the model could however mask potential differences at finer time scales such as higher evaporation from incipient cyanobacteria biocrusts particularly during the warm hours of the day (Chamizo et al., 2013a). Lichen biocrusts preserved more soil moisture despite lower infiltration rates due to their hydrophobic nature (Souza-Egipsy et al., 2002; Pintado et al., 2005) because they reduced of soil evaporation. The effect of biocrusts on evaporation is dynamic and can vary depending on soil properties and biocrust state (Chamizo et al., 2016a). For example, the water absorption by biocrust exopolysaccharides causes pore clogging and therefore reduces evaporation (Kidron et al., 1999; Belnap et al., 2005; Chamizo et al., 2013a; Rodríguez-Caballero et al., 2015b). Exopolysaccharide production, however, is variable over the year, therefore, evaporation measurements in the field can differ depending on when the sample was taken (Chamizo et al., 2013a, 2016c). In addition, biocrusts have also been shown to increase the water retention capacity of the uppermost soil layers, e.g., by increasing the amount of fine particles in the soil which also leads to higher soil moisture under biocrusts (Sun et al., 2021, 2022; Shi et al., 2023). Hence, the calibrated evaporation reduction factor in the model has likely captured additional biocrust effects on soil moisture that were not explicitly included in the model.

The observed vegetation distribution at the El Cautivo site reflects the simulated spatial pattern of water redistribution: Runoff from the biocrusted hillslope area provided additional water input to deeper soil layers in the middle part of the hillslope area that is mainly colonized by shrubs (Cantón et al., 2004). Our model results suggest that this water input is particularly important toward the end of the rain season that is still productive but already rather dry because. Additional water then allows for higher photosynthetic performance, carbon uptake rates and water-use efficiency (López-Ballesteros et al., 2018; Rodríguez-Caballero et al., 2018a). Such a positive effect of biocrust runoff on vascular vegetation through increased soil moisture has also been found in other drylands in Spain (Puigdefabregas et al., 1999), the Negev (Kidron and Aloni, 2018) and the Tengger desert (Li et al., 2008) and the positive effect of biocrusts on surface runoff emerges as a general pattern in drylands (Eldridge et al., 2020). The pediment of the El Cautivo hillslope is colonized by annual plants such as *Stipa capensis*, *Plantago ovata*, and *Bromus rubens* (Cantón et al., 2004). These plants lack the extensive rooting system of perennial plants to tap on deeper water sources which makes them more vulnerable to drought events within a season (Hamilton et al., 1999; Ruppert et al., 2015). However, they can evade these dry periods with dormancy which gives them an advantage over perennial plants in particularly dry sites. Therefore, as aridity increases, perennial vegetation abundance decreases while the cover of annual plants increases (Nunes et al., 2017), which can also be observed on the El Cautivo hillslope and supported by our simulation results. Lateral subsurface flow, which is not represented in the model, might further increase water redistribution in reality. At the El Cautivo site, soils in the upper part of the hillslope are shallow [0–2 cm of AC horizon followed by 2–30 cm C horizon (Cantón et al., 2003)] therefore the simulated deep drainage under biocrusts could in part be redistributed via subsurface flow toward downhill vegetated areas (Moore et al., 2015) which was also shown to be a substantial element of total runoff in a semi-arid hillslope in New Mexico (Wilcox et al., 1997).

### 4.2. Quantification of biocrust effects in a wet and dry year

Biocrusts positively affected overall hillslope soil moisture in both layers and in the wet and the dry year, highlighting their importance in preserving soil moisture for vascular plants. However, the degree of benefit varied across the hillslope and soil layers. In the wet season, biocrusts generally led to higher soil moisture in both layers, but in the dry season, these differences were small because soil moisture was close to the wilting point. In the upper soil layer, the protection from evaporation under biocrusts was the predominant driver of higher soil moisture. In the deeper layer this evaporation effect decreased, which has also been observed in the field (Chamizo et al., 2016c), instead, vegetation helped to balance reduced infiltration under biocrusts by increasing infiltration through preferential flow paths along the roots (Tietjen et al., 2009). There are also other factors that could in part explain the moisture differences between the layers that were not explicitly included in our model, but could be implicitly included in the calibrated parameter values. For example, field studies showed that shrubs can extend their rooting system below bare patches to harvest additional water from the deeper layers (Haase et al., 1996; Puigdefabregas et al., 1999). Also, biocrusts increase organic matter and soil water retention particularly in the first centimeter beneath the biocrust (Chamizo et al., 2012c) which can increase interlayer differences.

An exception to this wet season pattern is the month of December, where soil moisture was consistently higher in the bare soil scenario. In December, water input by dew is high (Uclés et al., 2014) and biocrusts are active and secreting high amounts of exopolysaccharides (Kappen and Valladares, 2007; Raggio et al., 2014; Chamizo et al., 2021). The pore clogging effect of exopolysaccharides can reduce infiltration of biocrusts and outweigh the benefits of lower evaporation (Kidron et al., 1999; Chamizo et al., 2013a,b). Higher infiltration rates in bare soils or after biocrust removal have also been reported in field studies (Xiao et al., 2019; Guan and Cao, 2021), particularly on dry soils (Chamizo et al., 2012b) but physical crust and incipient biocrusts quickly form and reduce infiltration again (Chamizo et al., 2012b).

Runoff was generally higher from biocrusts, except in December and March when bare soil had higher runoff. Incipient biocrusts and physical crusts have a lower surface roughness and can increase runoff velocity and shear strengths leading to rill formation (Rodríguez-Caballero et al., 2012). Runoff follows these preferential flow paths in open areas of the hillslope, increasing runoff connectivity, sediment and nutrient loss and thus reducing water and nutrient transfer to vegetated patches (Chamizo et al., 2016b). Although physical crusts can act as a water source to downstream vegetation (Cortina et al., 2010; Assouline et al., 2015), in highly erodible substrates, such as the marls of El Cautivo, significant amounts of sediment could be deposited under the plants in the long-term, further reducing the water and nutrient supply. These erosion and rill-formation processes are not included in the model, but are important for long-term hillslope development.

### 4.3. Difference in soil moisture and hydrological processes between well-developed and incipient biocrust

The different biocrust types had varying hydrological effects. Well-developed biocrusts, especially lichens, improved soil moisture by reducing evaporation and increasing deep drainage. Although lichen biocrusts can have lower infiltration capacities due to their hydrophobic behavior (Souza-Egipsy et al., 2002; Chamizo et al., 2012b), they can still improve underlying soil properties such as organic matter content, water retention, soil aggregation and porosity, leading to improved deep drainage and water retention below well-developed lichen and cyanobacteria biocrusts (Chamizo et al., 2012c; Felde et al., 2014; Cantón et al., 2020). This was also reflected in the modeled runoff, which was similar from all biocrust types, but the lichen biocrust yielded small runoff amounts when the other biocrusts infiltrated all rainfall water. Although these differences between biocrust types are relatively small, the effects accumulate and translate to the landscape scale leading to higher soil moisture in vegetated patches if the hillslope is covered with well-developed biocrusts (Supplementary Figure S12). This has also been found in other drylands where it was shown that even small losses of water from large biocrusted areas can provide substantial water to smaller runon zones and thus sustain islands of fertility for vascular plants (Kidron and Aloni, 2018).

Under climate change, the biocrust species composition is expected to shift toward earlier successional cyanobacteria biocrusts as studies from different drylands showed that lichens are particularly affected by rising temperatures (Escolar et al., 2012; Ladrón de Guevara et al., 2018; Baldauf et al., 2021; Finger-Higgens et al., 2022; Phillips et al., 2022). This shift is accompanied by an overall decrease in biocrust cover (Ladrón de Guevara et al., 2018; Rodríguez-Caballero et al., 2018b) which creates new open spaces of bare soil that are quickly replaced with physical crusts and incipient biocrusts (Chamizo et al., 2012b). These spaces can only be re-colonized by well-developed biocrusts in the long-term if soils are stable without major erosion events and rainfall is both sufficient and not too intense to destroy parts of the crust (Thomas and Dougill, 2007; Lázaro et al., 2008; Zhao et al., 2016; Cantón et al., 2020).

Our study site is covered with 40% biocrusts, 30% of which are lichens, therefore shifts in biocrust composition and cover will affect the ecohydrological processes and interactions between biocrusts and vegetated patches. The model results suggest that a shift toward incipient biocrusts or physical crust will lead to less plant-available water. Studies also showed that climate change, and warming in particular, impacts the role of biocrusts in hydrological processes both directly (via increasing evaporation) and indirectly (via altering the species composition) (Lafuente et al., 2018). Taken together, this can reduce perennial plant productivity and their capacity to effectively capture water (Ludwig et al., 2005; Puigdefábregas, 2005; Li et al., 2008; Rodríguez-Caballero et al., 2018a), thus increasing the potential for runoff flow connectivity (Rodríguez-Caballero et al., 2014a). Additionally, higher erosion rates and sediment loss are expected with reduced biocrust development compared to well-developed lichen biocrusts (Cantón et al., 2001, 2011; Belnap et al., 2013), especially for large rainfall events that fall on dry soils (Rodríguez-Caballero et al., 2012; Belnap et al., 2013; Belnap and Büdel, 2016), conditions that will become more frequent with increasing aridity in drylands across the globe (Huang et al., 2017). Ultimately, if soils are destabilized by this process, and biocrust recovery is not possible, a breakdown of the ecohydrological balance in dryland landscapes can be a result (Turnbull et al., 2012).

### 4.4. Using EcoHyD to model biocrust effects on dryland ecohydrology

In this study, we extended and used for the first time a spatially-explicit ecohydrological model (EcoHyD) to evaluate landscape-scale effects of biocrusts on water redistribution over the course of one year. Our extended EcoHyD model successfully reproduced soil moisture dynamics and water redistribution patterns for different biocrust types in South-East Spain. It has to be noted here, that we calibrated the model for the particularly wet hydrological year 2009–2010 with 63% more rainfall than the long-term average precipitation (374 mm vs. 235 mm average from 1967–1997, Lázaro et al. (2001)). Therefore, in the next step, it will be interesting to see if the calibrated parameters are robust and independent of the calibrated year and its rainfall. We identified the key processes governing soil moisture and water redistribution in a patchy dryland and assessed how they are affected by biocrusts using a process-based model. The EcoHyD model is developed in a general way making use of physical soil properties and hydrological process descriptions. This makes it suitable to apply to similar questions also in other drylands, as EcoHyd has already been successfully applied to drylands in the Middle East (Tietjen et al., 2009), Southern Africa (Lohmann et al., 2012; Guo et al., 2016; Irob et al., 2023) and China (Geissler et al., 2019). However, the effects of biocrust on evaporation and infiltration vary between regions and studies report a range of different results (e.g., with regard to evaporation see Chamizo et al., 2013a; Guan and Liu, 2019; Kidron et al., 2022). Therefore, the model needs to be carefully parameterized and validated for other regions before interpreting the results. Applying the model to different drylands worldwide could support the detection of general patterns of biocrust effects on landscape-scale water redistribution.

Moving forward, the model can be used to assess long-term hydrological dynamics and interactions between vegetated sink and biocrusted source areas in drylands. In our study, we simulated a single year and therefore did not expect significant changes in biocrust and vegetation cover. However, to study long-term dynamics, both biocrust and vegetation dynamics should be considered as their interactive feedbacks with soil properties and water dynamics control stabilizing mechanisms that lead to a heterogeneous landscape (Puigdefábregas, 2005; Turnbull et al., 2012). With such an extended model, these feedback could be explored and thresholds for irreversible damage to the ecohydrological balance, e.g., with regard to aridity or biocrust disturbance, could be identified.

## 5. Conclusion

In this study, we successfully included biocrusts into a spatial-explicit process-based ecohydrological model and simulated the landscape-scale processes of water redistribution and soil moisture for the first time in a spatially-explicit way. We found that areas covered with biocrusts effectively redistributed rainfall in the landscape leading to a differentiation in soil water availability. The redistribution process created a zone of higher water gain in the middle part of the hillslope which is colonized by shrubs and perennial vegetation in the landscape. Biocrusts increased water-availability to plants and potentially also landscape productivity. We found that biocrusts increased landscape-wide soil moisture during the rainy season (apart from December), a finding that cannot be detected in plot-scale field studies alone. Our simulation results showed that a climate change-induced shift from well-developed lichen to incipient cyanobacteria biocrusts could lead to lower soil moisture and less water redistribution in the future, which would also have consequences for vascular vegetation and the stability of the ecohydrological feedbacks on the landscape scale. The process-based model used in this study is a tool with which we can assess, quantify and upscale these observed processes and feedbacks and compare them in different scenarios of landscape configuration. The model has been well tested in other dryland areas where biocrusts are common. This opens the possibility to explore biocrust effects on landscape hydrology in these drylands as well. In a next step, the inclusion of dynamic vegetation, biocrusts and soil properties can allow us to investigate feedbacks and buffer mechanisms between source areas covered by biocrusts and sink areas covered by vascular vegetation. With such a model we can complement plot-based field studies and address questions on the resilience of dryland systems under climate change.

## Data availability statement

The datasets presented in this study can be found in online repositories. The names of the repository/repositories and accession number(s) can be found at: https://github.com/selinaZitrone/Baldauf_et_al_Biocrust_Ecohydrology.

## Author contributions

SB, BT, and YC planned and designed the research. SB conducted the modeling experiments, data analysis, and wrote the manuscript. YC provided the field data. All authors contributed to the data interpretation, revised the manuscript, and approved the submitted version.

## Funding

Funding was received through the Einstein Research Unit ‘Climate and Water under Change’ from the Einstein Foundation Berlin and Berlin University Alliance (ERU-2020-609). BT acknowledges funding by the German Research Foundation (DFG project TI 824/5-1). YC’s research was supported by the following projects: RH2O-ARID (P18-RT-5130) funded by the Junta de Andalucia with European Union funds for regional development; UAL2020-RNM-A2051, funded by the FEDER Andalucía 2014-2020 and Junta de Andalucía; and TED2021-132332B-C21 funded by MCIN/AEI/10.13039/501100011033 and European Unión “NextGenerationEU”/PRTR.

## Conflict of interest

The authors declare that the research was conducted in the absence of any commercial or financial relationships that could be construed as a potential conflict of interest.

## Publisher’s note

All claims expressed in this article are solely those of the authors and do not necessarily represent those of their affiliated organizations, or those of the publisher, the editors and the reviewers. Any product that may be evaluated in this article, or claim that may be made by its manufacturer, is not guaranteed or endorsed by the publisher.
