# Supplementary material for: Biocrusts intensify water redistribution and improve water availability to dryland vegetation: insights from a spatially-explicit ecohydrological model
Source: Front Microbiol. 2023 Jun 27;14:1179291. doi: 10.3389/fmicb.2023.1179291 (PMC10337590; doi:10.3389/fmicb.2023.1179291)
Supplement: Supplementary file 1 [file Data_Sheet_1.zip › Model description.docx]

Supplementary Material

Model description

In this study, we used the process-based ecohydrological dryland model EcoHyD and implemented a layer of biocrusts on top of the upper soil layer. The main model description of EcoHyD can be found in previous publications (hydrological submodel: Tietjen, Zehe, and Jeltsch (2009), vegetation sub-model: Lohmann et al. (2012); Tietjen et al. (2010)). biocrusts were implemented based on the model of Whitney et al. (2017)).

Biocrusts were realized as a layer on top of the first soil layer that mediates all water flows between the soil and the atmosphere. Figure 1 shows a flowchart of the main model processes and the order in which they are calculated.

# Hourly hydrological processes

## Rainfall

First, rainfall is converted into surface water ($water_{L0}$).

## Biocrust infiltration

Surface water can infiltrate into the biocrust until it is saturated. The potential biocrust infiltration ($I_{c}$, in $mm/h$) depends on the thickness of the biocrust layer ($Z_{c}$), the biocrust porosity ($n_{c}$) and the current biocrust moisture ($s_{c}$):

$$I_{c}=min\left( water_{L0}, Z_{c}*n_{c}*\left( 1-s_{c} \right) \right)$$

$I_{c}$ is multiplied with a hydrophobicity factor $f_{hyd}$ to account for water repellency of some biocrusts at low water contents. $f_{hyd}$ depends on two biocrust-specific parameters: The minimum value of the
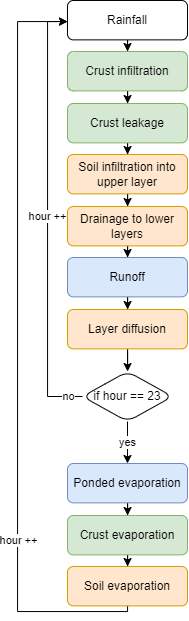
factor for a biocrust moisture of zero ($h_{min}$) and the critical biocrust water content $theta_{crit}$, at which the hydrophobicity factor becomes 1. Hydrophobicity increases exponentially between a biocrust moisture of 0 and $theta_{crit}$ (Figure 2):

$$f_{hyd}=h_{min}*e^{\left( \frac{-1}{theta_{crit}}*log\left( h_{min} \right)*s_{c} \right)}$$

The potential biocrust infiltration is multiplied with the hydrophobicity factor to determine the actual infiltration into the biocrust:

$$I_{c}=I_{c}*f_{hyd}$$

## Biocrust leakage

If the biocrust moisture exceeds the field capacity of the biocrust layer, surface water leaks into the upper soil layer.

The amount of water that leaks through the biocrust layer into the upper soil layer depends on the saturated hydraulic conductivity of the biocrust ($K_{s,c}$), the pore size distribution ($\beta_{c}$) and the field capacity ($s_{fc,c}$) of the biocrust layer:

$$L_{c}=K_{s,c}\left( \frac{e^{\beta_{c}\left( s_{c}-s_{fc,c} \right)}-1}{\beta_{c}\left( 1-s_{fc,c} \right)-1} \right)$$

If fully saturated, leakage is maximal and limited by the saturated hydraulic conductivity of the biocrust. The pore size distribution $\beta_{c}$ is calculated from the pore distribution parameter $b_{c}$:

**Figure 1:** Flowchart of model processes. Biocrust processes are shown in green, soil processes in orange and surface processes in blue.

$$\beta_{c}=2*b_{c}+4$$

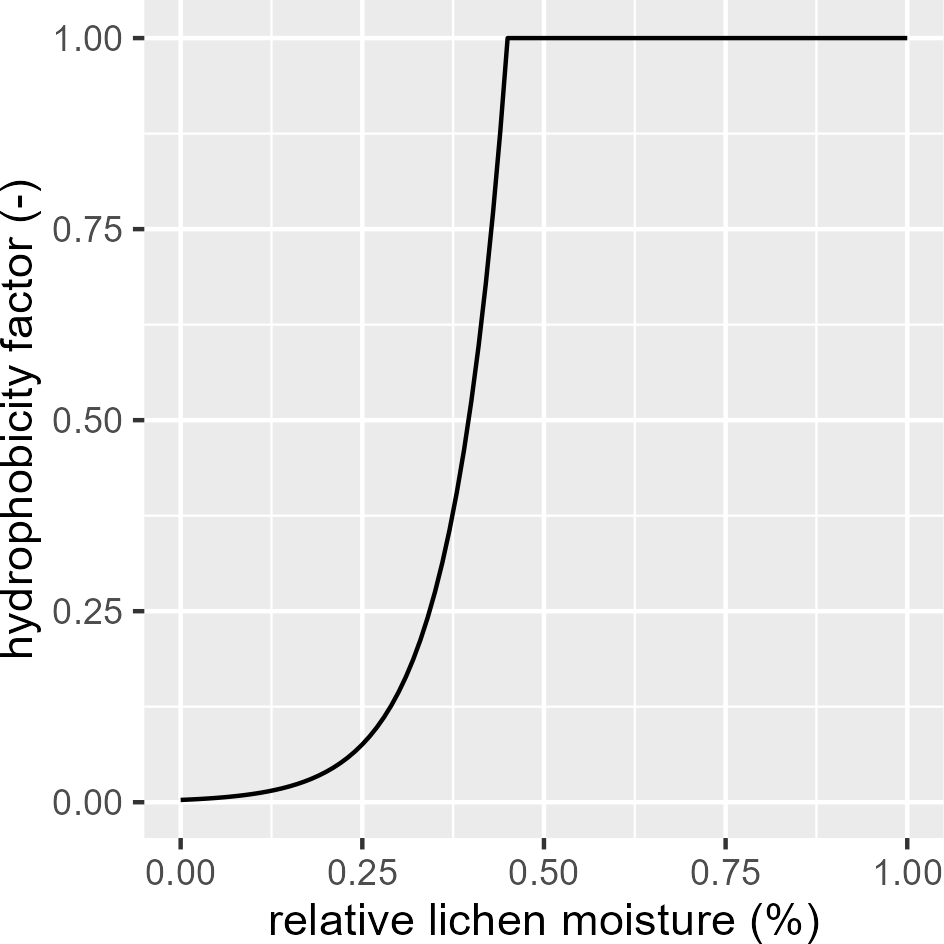


**Figure 2:** Values for hydrophobicity factor $f_{hyd}$ at different lichen moisture values.

## Soil infiltration upper layer

The water that leaks through the biocrust can infiltrate into the upper soil layer according to the Green and Ampt approach (Green and Ampt 1911), where a wetting front proceeds through the soil. Above the wetting front, the soil is saturated, below it is not. See Tietjen, Zehe, and Jeltsch (2009) for further information on the soil infiltration in EcoHyD. If not all the leaked water could infiltrate into the soil in this time step, ponding occurs. The ponded water is added back to the surface water $water_{L0}$.

## Drainage to lower layers

If after soil infiltration, the soil moisture in the upper layer exceeds its field capacity, the excess water drains into the lower soil layer. If also the lower soil layer exceeds field capacity after drainage from the upper soil layer, excess water drains from the lower soil layer.

## Runoff

The remaining surface water is available for runoff. Runoff is calculated following a Manning-Strickler approach (Dingman 1994; Tietjen, Zehe, and Jeltsch 2009):

$$QD=water_{L0}^{\frac{2}{3}}*\sqrt{sl}*\left( 1-\frac{c_{g}+c_{s}}{2} \right)*f_{c}$$

with

- $water_{L0}$: surface water [mm]
- $sl$: downward slope of the cell (lowest neighbouring cell)
- $c_{g}$ and $c_{s}$: vegetation cover of grasses and shrubs
- $f_{c}$: cover of biocrust

$f_{c}$ was taken to be 3.6 in this study because measurements at the research site showed that runoff from biocrusted soil on average is 3.6 times higher than on non-biocrusted soils (Cantón et al. 2002; 2001).

Diffusion happens between the upper and the lower soil layer to balance differences in moisture between the layers. Diffusion follows Darcy’s law, adopted for soil moisture (see description in Tietjen, Zehe, and Jeltsch (2009)) and depends on the difference in soil moisture between layers, the geometric mean of saturated and unsaturated hydraulic conductivity, and a dimensionless diffusion constant (Tietjen, Zehe, and Jeltsch 2009).

# Daily hydrological processes

Evaporation from the surface water and the biocrusts layer and evapotranspiration from the soil layers is calculated once at the end of every day.

## Potential evapotranspiration

First, the potential evapotranspiration demand of the given day is calculated ($ET_{pot}$). Potential evapotranspiration is calculated based on Hargreaves (1974). It depends on the daily mean ($\underline{T}$), minimum ($T_{min}$) and maximum ($T_{max}$) temperature and the extraterrestrial radiation ($R_{ext}$) on that day (estimated from the latitude of the site and the Julian day ($J$)).

$$ET_{pot}=0.0023*\left( \underline{T}+17.8 \right)*\left( T_{max}-T_{min} \right)^{0.5}*R_{ext}$$

with

$$R_{ext}=15.392*d_{r}*\left( \omega_{s}*sin\phi*sin\delta*sin\omega_{s} \right)$$

with

- $d_{r}=1+0.033*cos\left( \frac{2\pi}{365}*J \right)$: distance between earth and sun
- $\omega_{s}=arccos\left( -tan\phi*tan\delta\right)$: sunset hour angle
- $\phi=\frac{lat*\pi}{180}$: latitude of site in radian
- $\delta=0.4093*sin\left( \frac{2\pi}{365}*J-1.405 \right)$: solar declination in radian.

$ET_{pot}$ is then sequentially met by evaporation of ponded surface water (free evaporation of all surface water), biocrust evaporation and soil evapotranspiration.

## Biocrust evaporation

Water can only evaporate from the biocrust if biocrust moisture is above the hygroscopic point ($s_{h,c} \left( - \right)$), otherwise it is bound to the biocrust layer (Whitney et al. 2017). Evaporation from the biocrust layer is represented as a fraction $k_{c}$ of the potential evaporation which is a parameter depending on the level of development of the biocrust.

$$E_{c}\left( s_{c} \right)=\left\{ \begin{aligned} 0 \\ k_{c}*PET \end{aligned} \right. {s_{c}\leq s_{h,c} \atop s_{c}>s_{h,c}}$$

with

- $s_{h,c} \left( - \right)$: hygroscopic point of biocrust layer
- $k_{c} \left( - \right)$: weighting term to limit biocrust evaporation

If the evaporation of the biocrust exceeds the remaining evaporative demand, the remaining demand is met and the rest of the water remains within the biocrust layer. Otherwise, the remaining evaporative demand is reduced by the amount of water evaporated from the biocrust layer.

## Soil evapotranspiration

Soil evaporation adopts a conceptual HBV model, similar to other ecohydrological models. If soil moisture is above the wilting point, evaporation is maximal and depends only on the vegetation cover and a factor that reduces evaporation due to soil crusting. Between the residual water content ($s_{res}$) and the wilting point ($WP$), soil evaporation increases exponentially (Figure 3).

$$E_{s}=\left\{ \begin{aligned} min\left( ET_{pot}*cosincl*vegfunction*f_{crust},\left( s-s_{res} \right)*Z_{s} \right) \\ max\left( 0,min\left( ET_{pot}*\left( s/WP \right)^{2}*vegfunction*f_{crust},\left( s-s_{res} \right)*Z_{s} \right) \right) \end{aligned} \right.{s>WP \atop s\leq WP}$$

with

- $ET_{pot}$: potential evaporation (here: remaining evaporative demand after ponded and biocrust evaporation)
- $incl$: inclination of the current cell
- $vegfunction$
  - in layer 1: $vegfunction=c_{vegET_{1}}-c_{vegET_{2}}*\left( cover_{g}+cover_{a}+cover_{s} \right)$
    - $c_{vegET_{1}}$ & $c_{vegET_{1}}$ : Coefficients determining the influence of vegetation cover on evapotranspiration due to shading effects
  - in layer 2: $P_{totroot_{g}}+P_{totroot_{s}}$
    - $P_{totroot}=f_{root}*cover$ with $f_{root}$ being a parameter determining the conversion of surface cover of a pft to the root fraction in the specific layer
    -
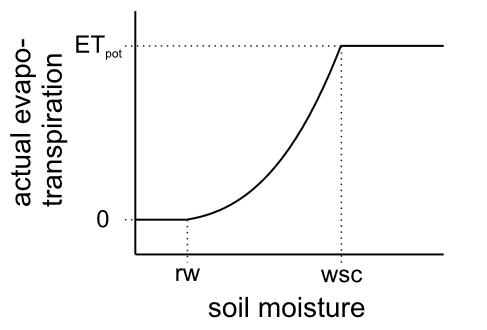
Total roots calculated for both perennial grasses $P_{totroot_{g}}$ and shrubs $P_{totroot_{s}}$

**Figure 3:** Conceptual overview of actual evapotranspiration $E_{s}$ depending on soil moisture. The actual evapotranspiration increases exponentially between the residual water content $rw$ and the wilting point $wsc$. Figure from Tietjen, Zehe, and Jeltsch (2009)

- $f_{crust}$: factor reducing soil evaporation due to presence of biocrusts (depends on biocrust type)
- $s$: soil moisture
- $s_{res}$: residual soil moisture
- $Z_{s}$: depth of soil layer
- $WP$: wilting point

# References

Cantón, Yolanda, Francisco Domingo, A. Solé-Benet, and J. Puigdefábregas. 2002. ‘Influence of Soil-Surface Types on the Overall Runoff of the Tabernas Badlands (South-East Spain): Field Data and Model Approaches’. *Hydrological Processes* 16 (13): 2621–43. https://doi.org/10.1002/hyp.1052.

Cantón, Yolanda, Francisco Domingo, A. Solé-Benet, and Juan Puigdefábregas. 2001. ‘Hydrological and Erosion Response of a Badlands System in Semiarid SE Spain’. *Journal of Hydrology* 252: 65–84.

Dingman, SL. 1994. *Physical Hydrology*. New York: Macmillan.

George H. Hargreaves. 1974. ‘Estimation of Potential and Crop Evapotranspiration’. *Transactions of the ASAE* 17 (4): 0701–4. https://doi.org/10.13031/2013.36941.

Green, Heber W., and G. A. Ampt. 1911. ‘Studies on Soil Physics’. *The Journal of Agricultural Science* 4 (1): 1–24.

Lohmann, Dirk, Britta Tietjen, Niels Blaum, David F. Joubert, and Florian Jeltsch. 2012. ‘Shifting Thresholds and Changing Degradation Patterns: Climate Change Effects on the Simulated Long-Term Response of a Semi-Arid Savanna to Grazing’. *Journal of Applied Ecology* 49 (4): 814–23. https://doi.org/10.1111/j.1365-2664.2012.02157.x.

Tietjen, Britta, Florian Jeltsch, Erwin Zehe, Nikolaus Classen, Alexander Groengroeft, Katja Schiffers, and Jens Oldeland. 2010. ‘Effects of Climate Change on the Coupled Dynamics of Water and Vegetation in Drylands’. *Ecohydrology*, n/a-n/a. https://doi.org/10.1002/eco.70.

Tietjen, Britta, Erwin Zehe, and Florian Jeltsch. 2009. ‘Simulating Plant Water Availability in Dry Lands under Climate Change: A Generic Model of Two Soil Layers’. *Water Resources Research* 45 (1): 1–14. https://doi.org/10.1029/2007WR006589.

Whitney, Kristen M., Enrique R. Vivoni, Michael C. Duniway, John B. Bradford, Sasha C. Reed, and Jayne Belnap. 2017. ‘Ecohydrological Role of Biological Soil Crusts across a Gradient in Levels of Development’. *Ecohydrology* 10 (7): e1875–e1875. https://doi.org/10.1002/eco.1875.
