# Supplementary material for: Biocrusts intensify water redistribution and improve water availability to dryland vegetation: insights from a spatially-explicit ecohydrological model
Source: Front Microbiol. 2023 Jun 27;14:1179291. doi: 10.3389/fmicb.2023.1179291 (PMC10337590; doi:10.3389/fmicb.2023.1179291)
Supplement: Supplementary file 1 [file Data_Sheet_1.zip › Supplementary Figures.docx]

Supplementary Figures

Biocrusts intensify water redistribution and improve water availability to dryland vegetation: insights from a spatially-explicit ecohydrological model

Selina Baldauf*, Yolanda Cantón, Britta Tietjen

*** Correspondence:** Corresponding Author: selina.baldauf@fu-berlin.de


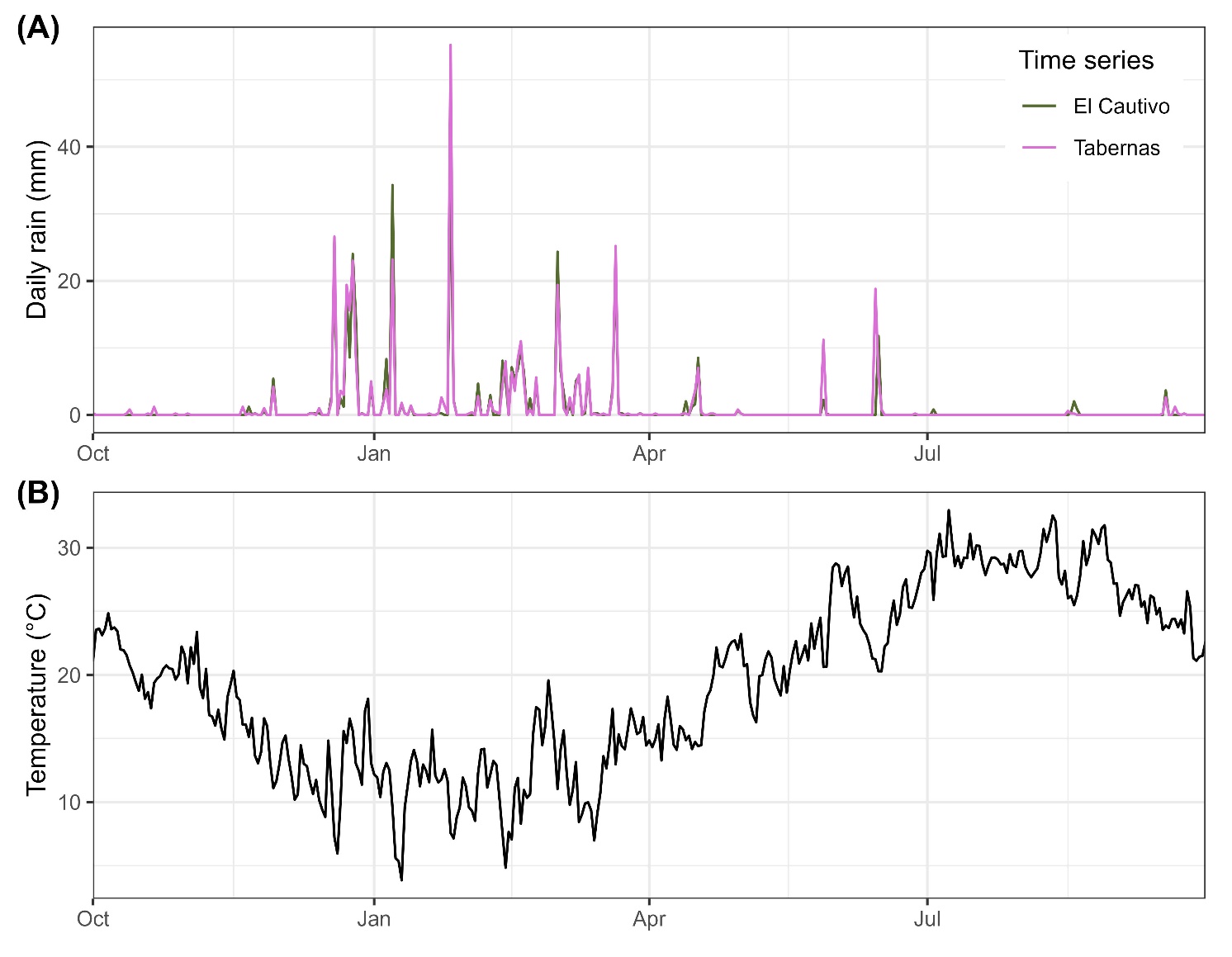


**Supplementary Figure 1.** Climate in El Cautivo. **(A)** Comparison of daily rainfall sum from the El Cautivo and the Tabernas weather station. Climate in El Cautivo. **(B)** Time series of daily mean temperatures. Time series was reordered from the hydrological year October 2009 - September 2010 and used as model input.

**
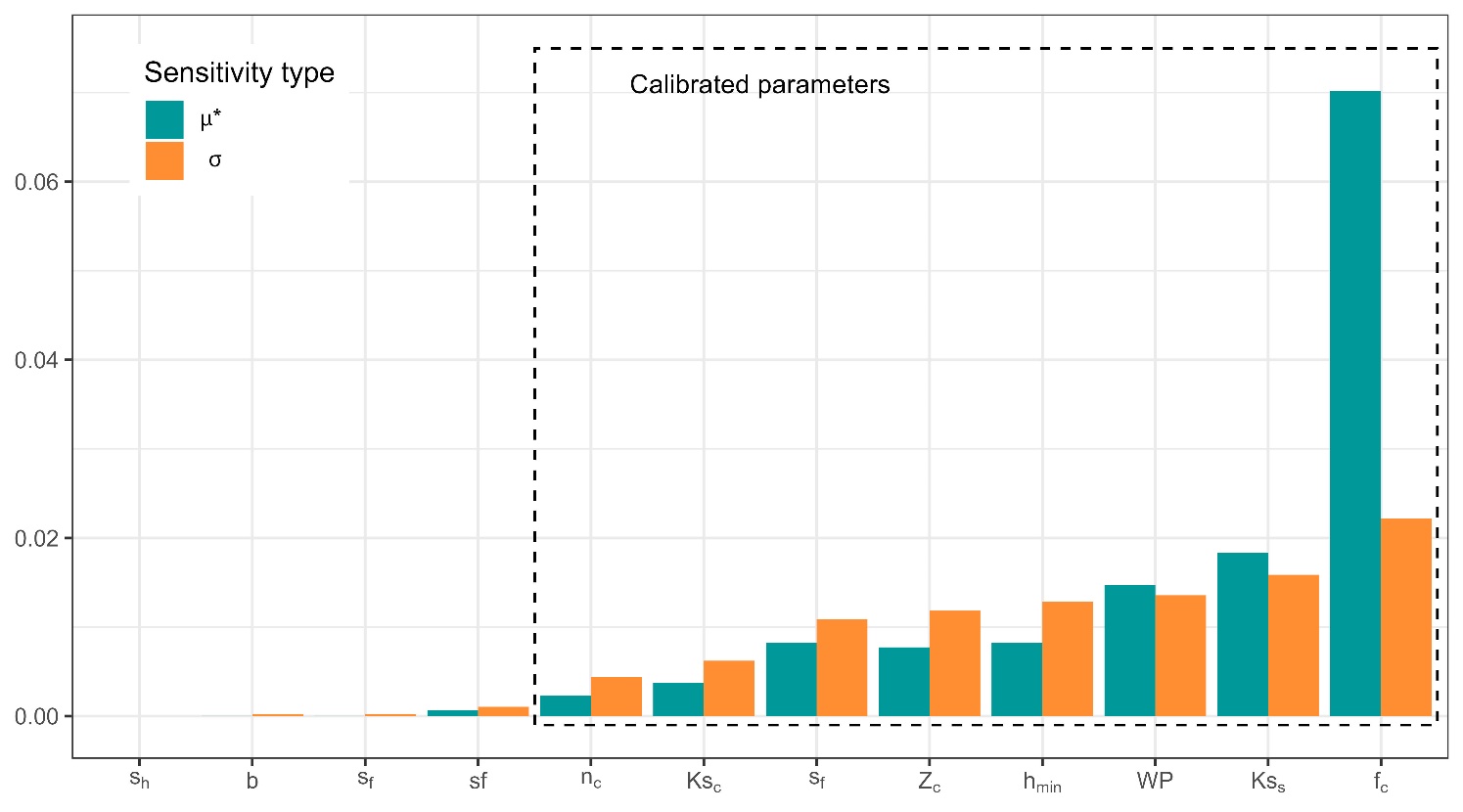
**

**Supplementary Figure 2.** Results of Morris screening for the target function of the calibration (RMSE of observed and modelled soil moisture in 3 and 10 cm depth). Soil parameters are written in blue font and biocrust parameters in red font. The parameters in the black rectangle on the right are used for calibration. µ* represents the overall influence of a factor (mean of distribution) and shows the non-linear/interactive effects of a factor (standard deviation of the distribution).


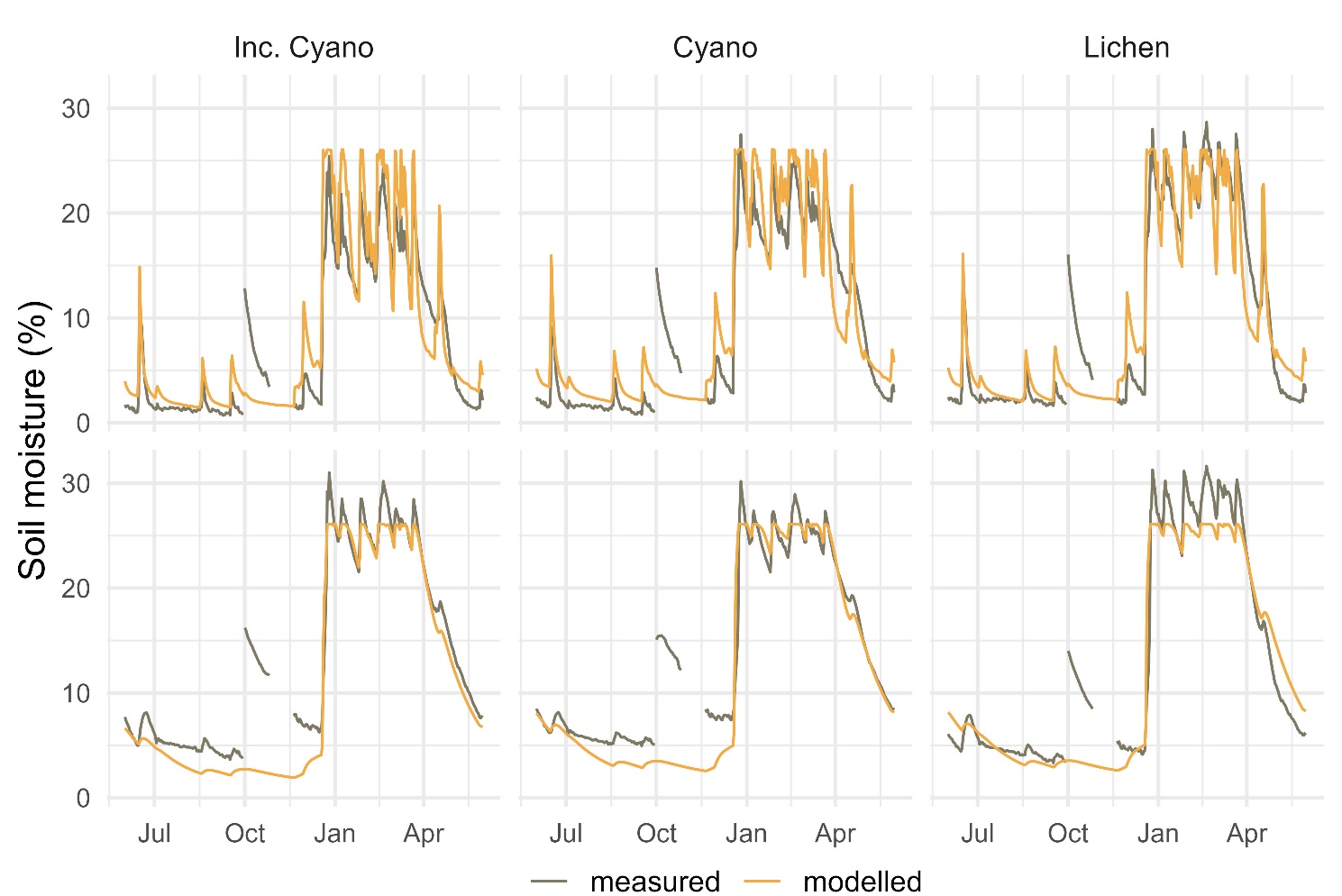


**Supplementary Figure 3.** Comparison of modelled and measured soil moisture under the three biocrust types for the calibrated parameter set in the upper (top row) and the deeper (bottom row) layer. Calibration period were the months of December and January.


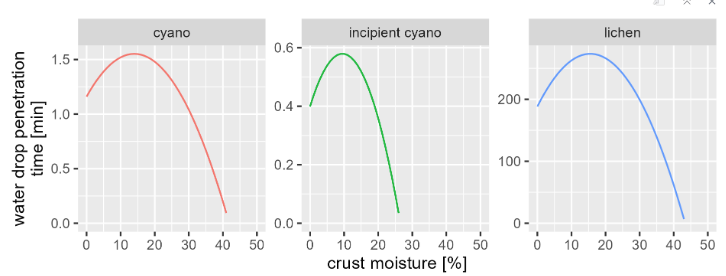


**Supplementary Figure 4.** Quadratic fit of water drop penetration time measured in the lab for 3 different biocrust types.


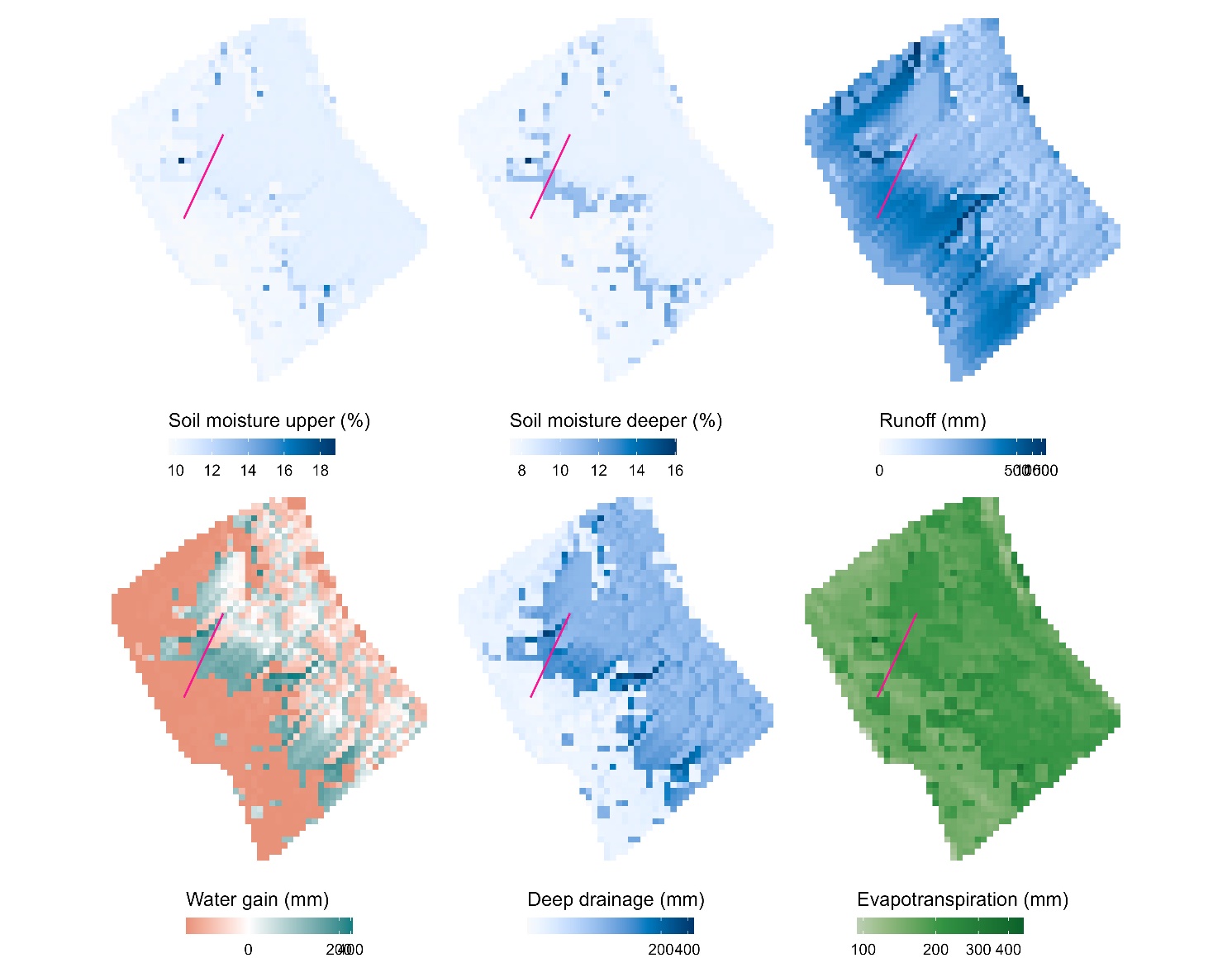


**Supplementary Figure 5.** Mean monthly soil moisture and monthly sum of hydrological processes for each grid cell on the El Cautivo hillslope in December. The pink line shows the hillslope transect that was selected for further detailed spatio-temporal analysis. It crosses a biocrusted section at the top (SW) and a vegetated section further down the hillslope (see also Fig. 1).


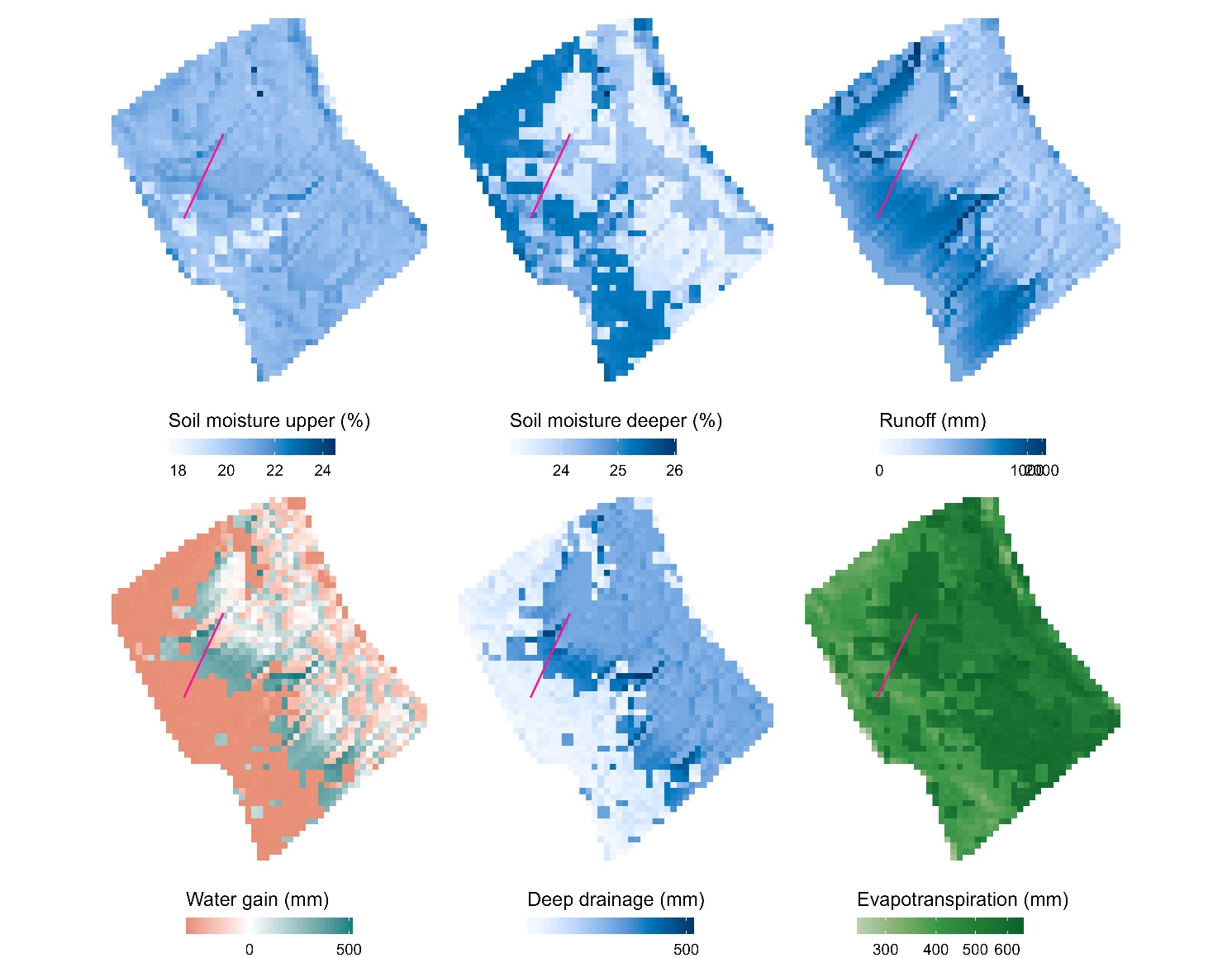


**Supplementary Figure 6.** Mean monthly soil moisture and monthly sum of hydrological processes for each grid cell on the El Cautivo hillslope in January. The pink line shows the hillslope transect that was selected for further detailed spatio-temporal analysis. It crosses a biocrusted section at the top (SW) and a vegetated section further down the hillslope (see also Fig. 1).


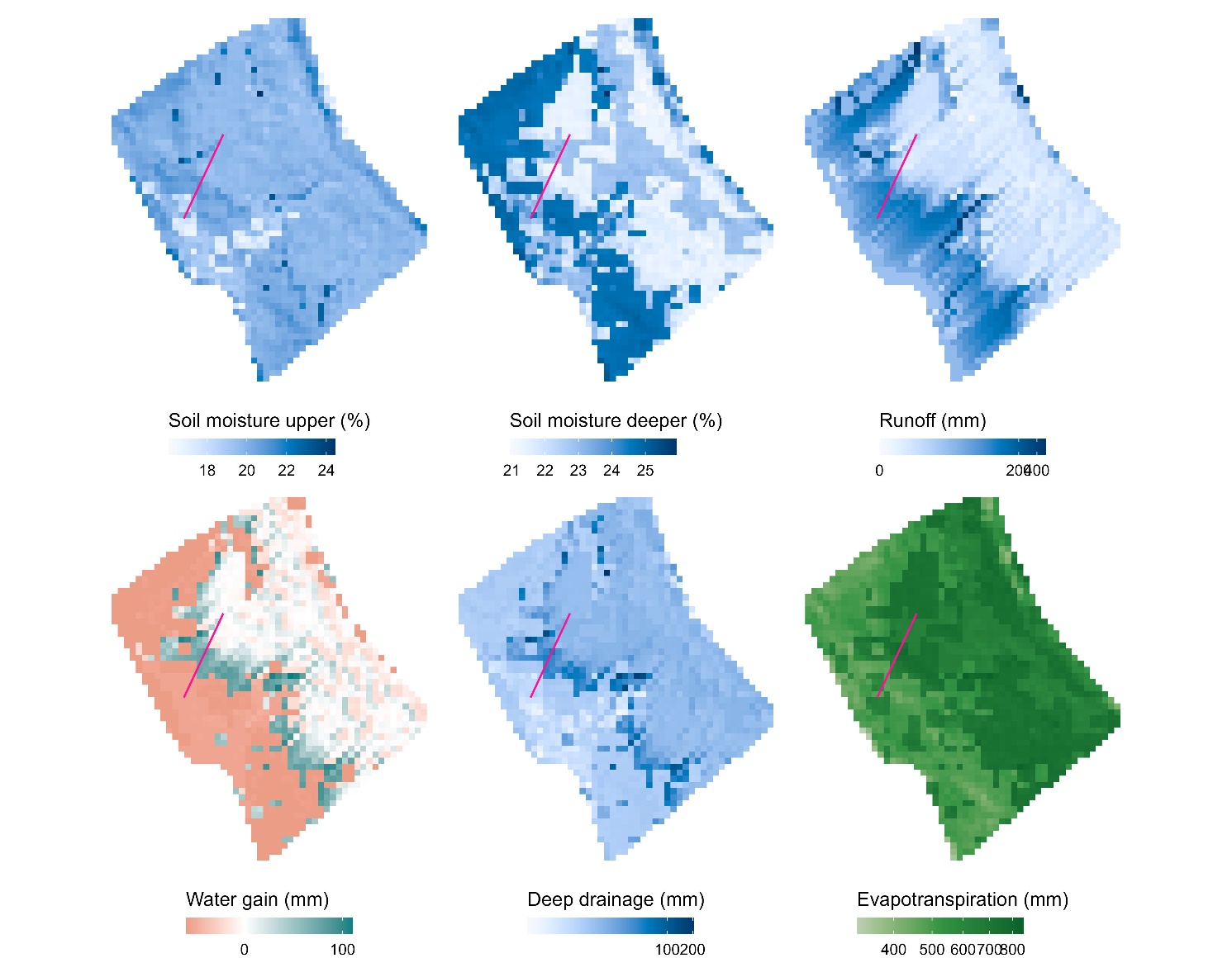


**Supplementary Figure 7.** Mean monthly soil moisture and monthly sum of hydrological processes for each grid cell on the El Cautivo hillslope in February. The pink line shows the hillslope transect that was selected for further detailed spatio-temporal analysis. It crosses a biocrusted section at the top (SW) and a vegetated section further down the hillslope (see also Fig. 1).


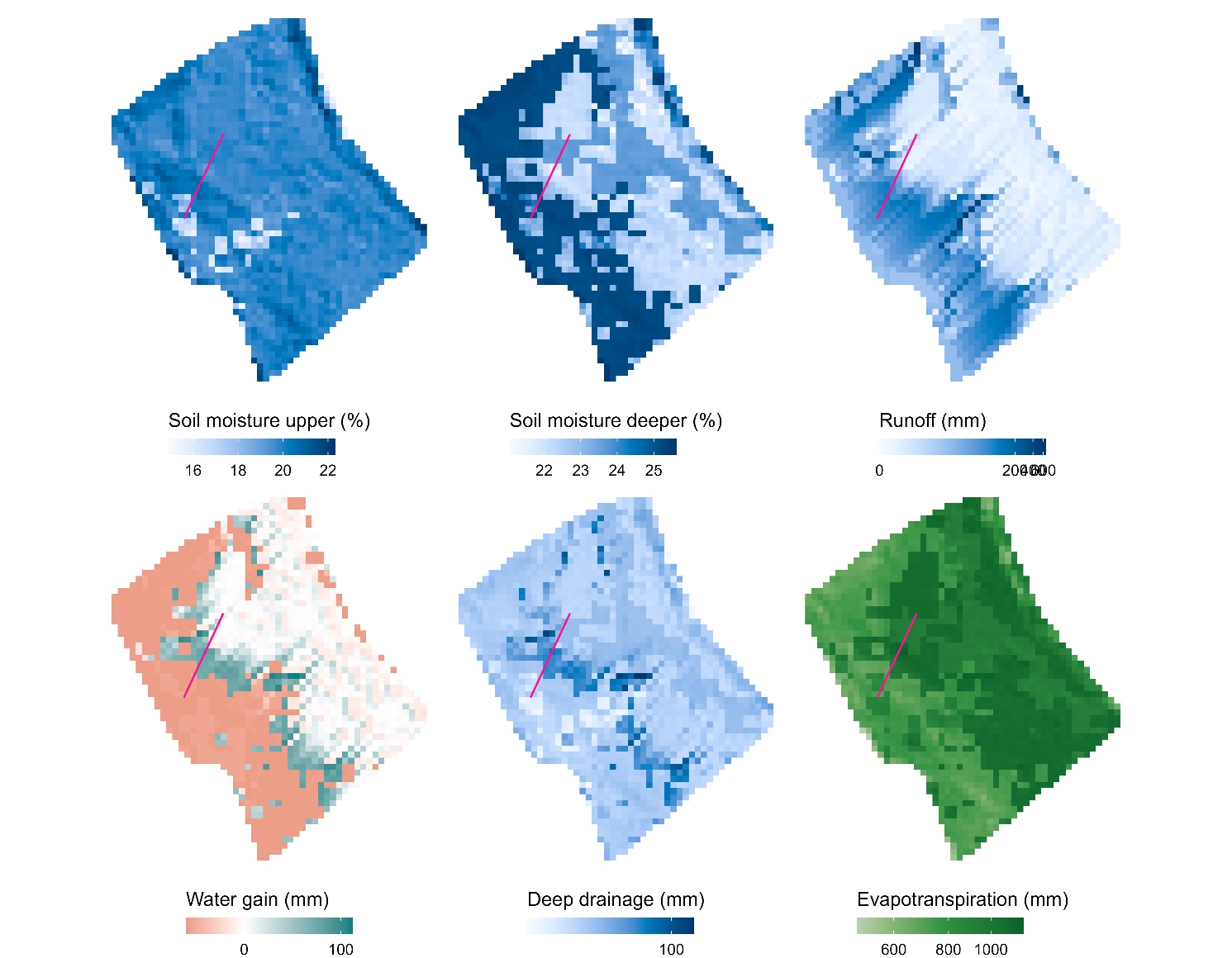
**Supplementary Figure 8.** Mean monthly soil moisture and monthly sum of hydrological processes for each grid cell on the El Cautivo hillslope in March. The pink line shows the hillslope transect that was selected for further detailed spatio-temporal analysis. It crosses a biocrusted section at the top (SW) and a vegetated section further down the hillslope (see also Fig. 1).

**
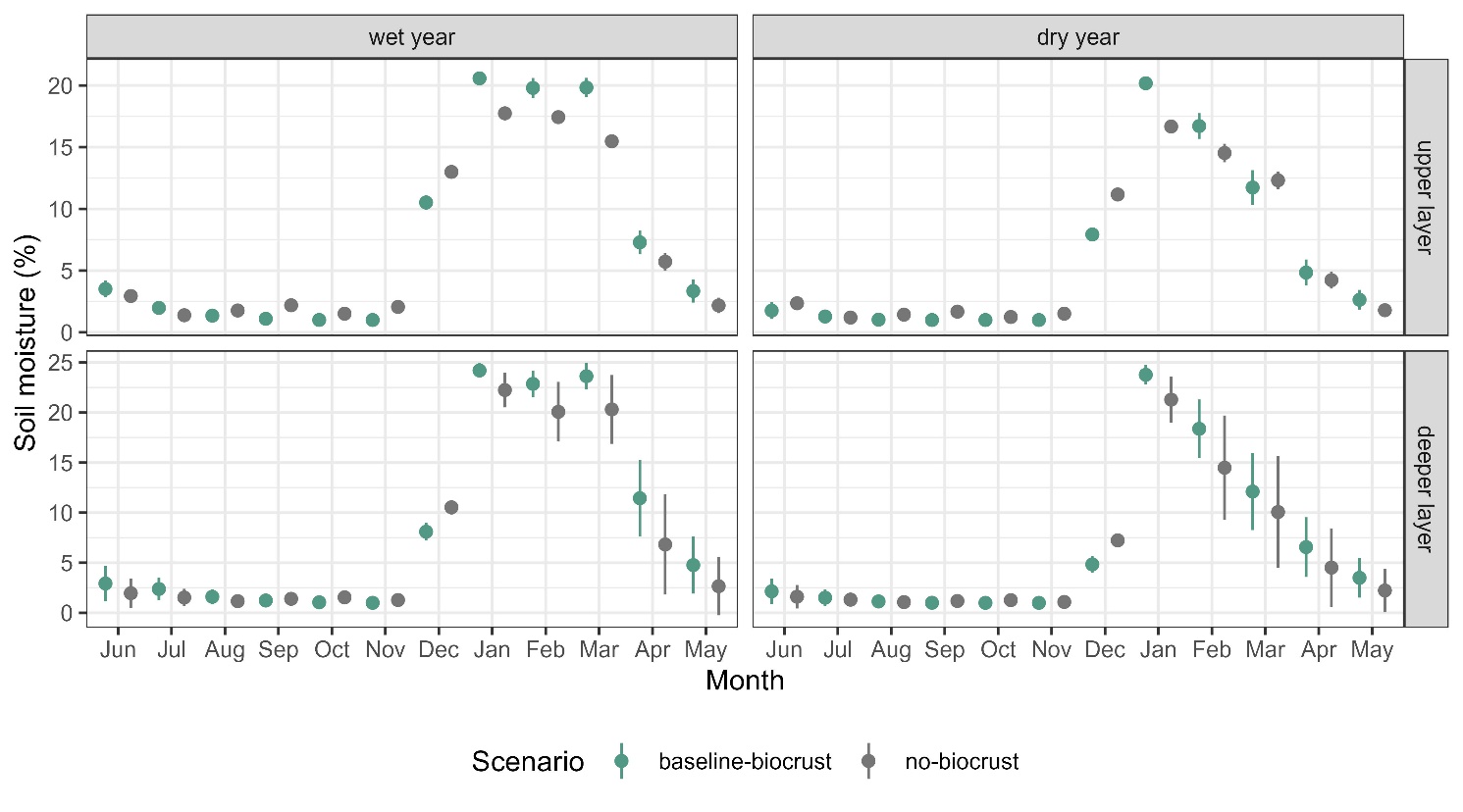
Supplementary Figure 9.** Effect of biocrusts on soil moisture in a wet and a dry year. Distribution of mean monthly soil moisture in the upper and lower layer of all grid cells of the hillslope between the baseline-biocrust scenario with and the no-biocrust scenario without biocrust cover. The first two column shows the distribution in a wet year and the second column shows the distribution in a dry year with 50% reduced rainfall.


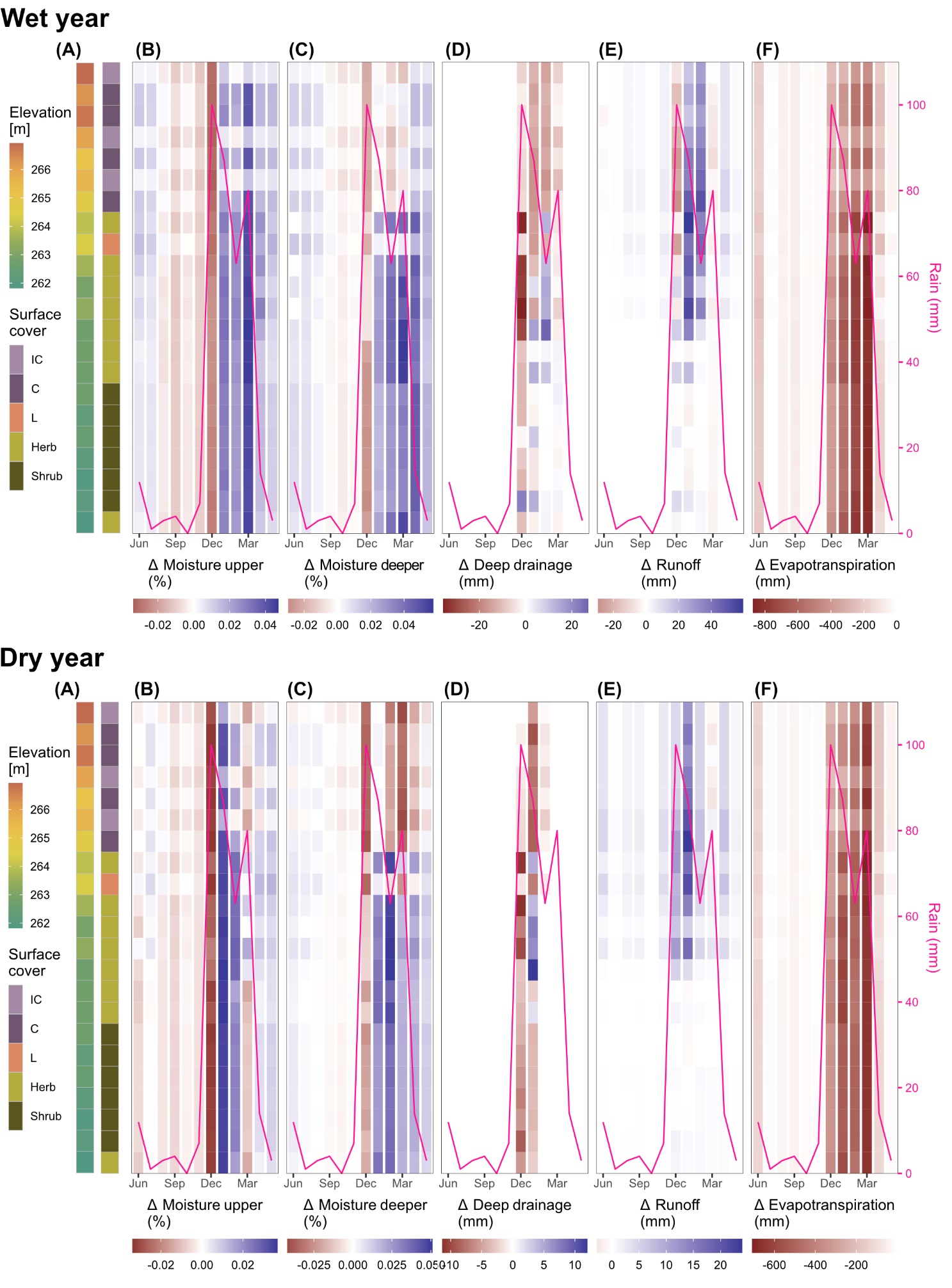


**Supplementary Figure 10.** Impacts of the presence of biocrusts on water availability and fluxes along the transect for a wet year (top) and a dry year (bottom). **(A)**: Elevation and surface cover of the transect cells selected for the spatio-temporal analysis. See Fig. 1 B-D for location of the transect in the hillslope. **(B-F)**: Spatio-temporal development of the absolute differences between the baseline-biocrust scenario with and the no-biocrust scenario without biocrusts. Differences are calculated by subtracting mean monthly soil moisture or monthly sums of deep drainage, runoff and evapotranspiration of the no-biocrust scenario from the baseline-biocrust scenario. Therefore, red colours indicate cells in which the value is lower in the baseline-biocrust scenario and blue colours indicate cells in which the values were higher in the baseline-biocrust compared to the no-biocrust scenario. The figures show these differences for each transect cell (y-axis) over the course of one year (x-axis).


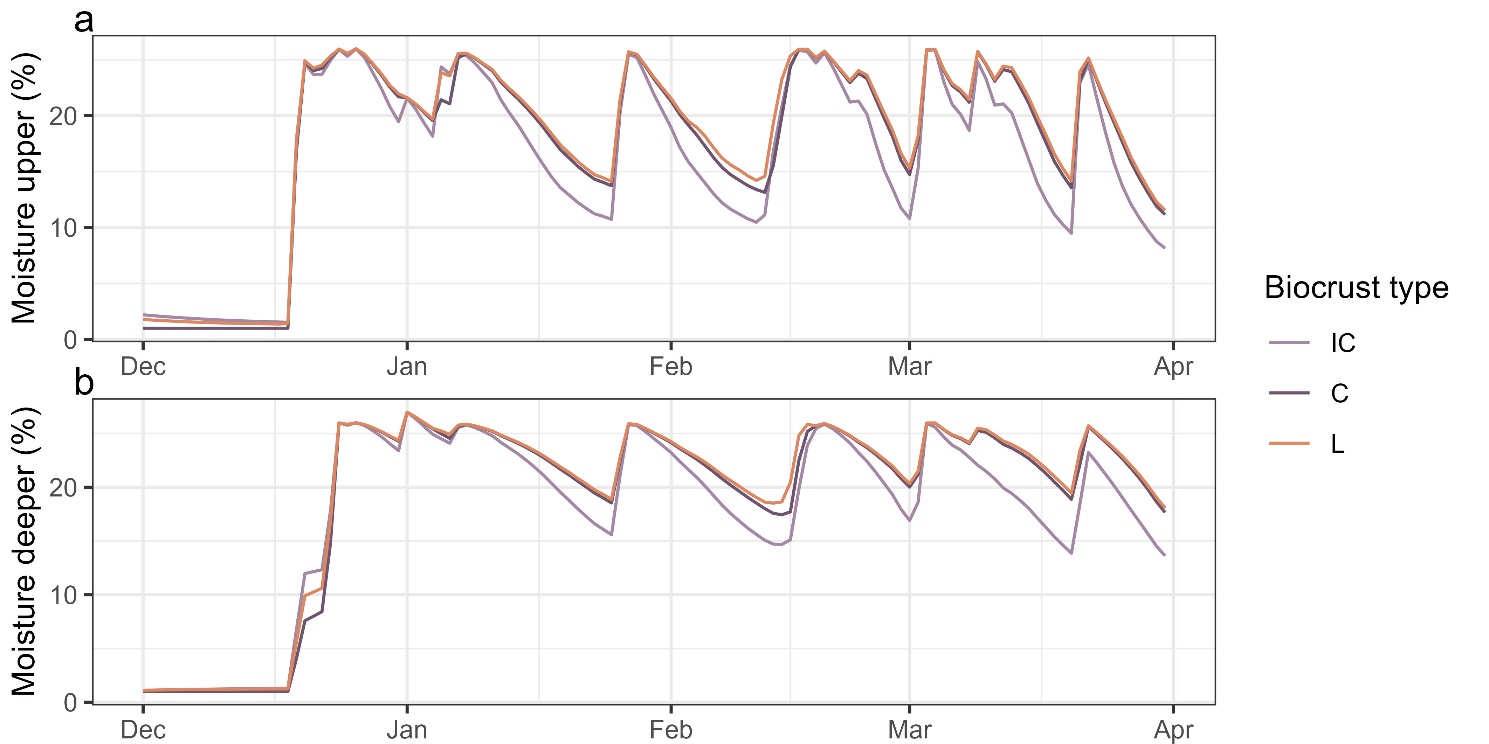


**Supplementary Figure 11.** Comparison of the effect of different biocrust types (IC - incipient cyanobacteria, C - cyanobacteria, L - lichen) on soil moisture in the vegetated hillslope cells in the wet season from January until March.
