# Supplementary material for: Biocrusts intensify water redistribution and improve water availability to dryland vegetation: insights from a spatially-explicit ecohydrological model
Source: Front Microbiol. 2023 Jun 27;14:1179291. doi: 10.3389/fmicb.2023.1179291 (PMC10337590; doi:10.3389/fmicb.2023.1179291)
Supplement: Supplementary file 1 [file Data_Sheet_1.zip › Supplementary Tables.DOCX]

Supplementary Tables

Biocrusts intensify water redistribution and improve water availability to dryland vegetation: insights from a spatially-explicit ecohydrological model

Selina Baldauf*, Yolanda Cantón, Britta Tietjen

*** Correspondence:** Corresponding Author: selina.baldauf@fu-berlin.de

Supplementary Table 1. Parameters and the value ranges used in the sensitivity analysis and calibration.

| **Parameter** | **Symbol** | **Unit** | **Lower bound** | **Upper bound** | **References** | **Note** | **Calibration** |
| --- | --- | --- | --- | --- | --- | --- | --- |
| **Crust parameters** | | | | | | | |
| Porosity | *n_c_* | - | 0.3  (0.3, 0.3, 0.3) | 0.5  (0.5, 0.5, 0.5) | (Whitney et al. 2017) | No measurements available, used values within range reported in literature | yes |
| Thickness | *Z_c_* | mm | 1  (1, 1, 5) | 20  (10, 15, 20) |  | No measurements available, used range reported in literature | yes |
| Saturated hydraulic conductivity | *Ks_c_* | mm/h | 2.7  (9.5, 9.5, 2.7) | 25.9  (20.9, 25.9, 15.1) |  | Range of mean ± standard deviation of Ks reported under different biocrust types | yes |
| Evaporation reduction factor | *f_c_* | - | 0.1  (0.1, 0.1, 0.1) | 1  (1, 1, 1) |  | - | yes |
| Minimum hydrophobicity factor | *h_min_* | - | 0.01 | 1 |  | Only calibrated for lichen biocrust | yes |
| Pore distribution parameter | b | - | 2 | 3.5 |  | - | no |
| Crust field capacity | *s_fc,c_* | - | 0.2 | 0.8 |  | - | no |
| Hygroscopic point | *s_h,c_* | - | 0.08 | 0.44 |  | - | no |
| **Soil parameters** | | | | | | | |
| Suction at the wetting front | *sf* | mm | 133 | 201 |  | No measurement available, but standard value for silty loam is 166.8 in EcoHyD. Here we took this value and tested sensitivity within a range of ±20% | no |
| Saturated hydraulic conductivity | *Ks_s_* | mm/h | 5 | 20 |  | Range of mean ± standard deviation | yes |
| Wilting point | WP | - | 0.08 | 0.18 |  | Range taken from reported values in literature and the standard value for silty loam in EcoHyD | yes |
| Field capacity | *s_fc,s_* | - | 0.25 | 0.31 |  | Range taken from reported value in literature and limited by the maximum soil moisture observed in the data | yes |

*Note:* If the 3 upper and lower bound values in parentheses refer to the upper and lower bound of incipient cyanobacteria, cyanobacteria and lichen biocrust, respectively (Whitney et al. 2017).

Supplementary Table 2. Soil parameter values used in the model.

| **Parameter** | **Symbol** | **Unit** | **Value** | **Reference** |
| --- | --- | --- | --- | --- |
| Suction at the wetting front | *sf* | mm | 166 | (Tietjen, Zehe, and Jeltsch 2009) |
| Saturated hydraulic conductivity | *Ks_s_* | mm/h | 6.9 | Calibration |
| Wilting point | WP | - | 0.18 | Calibration |
| Field capacity | *sf_c,s_* | - | 0.26 | Calibration |
| Saturation water content | *s_s,max_* | - | 0.26 | Same as field capacity |
| Depth upper layer | *Z_s1_* | mm | 60 | From data (soil moisture measurements) |
| Depth lower layer | *Z_s2_* | mm | 80 | From data (soil moisture measurements) |
| Residual water content | *s_s,res_* | - | 0.01 | From data (soil moisture measurements) |
| Diffusion constant | *c_diff_* | - | 0.05 | (Tietjen, Zehe, and Jeltsch 2009) |

Supplementary Table 3. Biocrust parameter values used in the model.

| **Parameter** | **Symbol** | **Unit** | **Inc. cyanobacteria** | **Cyanobacteria** | **Lichen** | **Reference** |
| --- | --- | --- | --- | --- | --- | --- |
| Porosity | *n_c_* | - | 0.5 | 0.5 | 0.38 | Calibration |
| Thickness | *Z_c_* | mm | 10 | 16.6 | 17.2 | Calibration |
| Saturated hydraulic conductivity | *Ks_c_* | mm/h | 17.9 | 17 | 8.9 | Calibration |
| Evaporation reduction factor | *fc* | - | 0.6 | 0.4 | 0.38 | Calibration |
| Minimum hydrophobicity factor | *h_min_* | - | no hydrophob. | no hydrophob. | 0.47 | Calibration |
| Pore distribution parameter | b | - | 3.1 | 2.8 | 2.5 | (Whitney et al. 2017) |
| Crust field capacity | *s_fc,c_* | - | 0.27 | 0.28 | 0.47 | (Whitney et al. 2017) |
| Hygroscopic point | *s_h,c_* | - | 0.08 | 0.09 | 0.12 | (Whitney et al. 2017) |

**References**

Tietjen, Britta, Erwin Zehe, and Florian Jeltsch. 2009. ‘Simulating Plant Water Availability in Dry Lands under Climate Change: A Generic Model of Two Soil Layers’. *Water Resources Research* 45 (1): 1–14. https://doi.org/10.1029/2007WR006589.

Whitney, Kristen M., Enrique R. Vivoni, Michael C. Duniway, John B. Bradford, Sasha C. Reed, and Jayne Belnap. 2017. ‘Ecohydrological Role of Biological Soil Crusts across a Gradient in Levels of Development’. *Ecohydrology* 10 (7): e1875–e1875. https://doi.org/10.1002/eco.1875.
